# A dual-targeting approach to inhibit *Brucella abortus* replication in human cells

**DOI:** 10.1038/srep35835

**Published:** 2016-10-21

**Authors:** Daniel M. Czyż, Neeta Jain-Gupta, Howard A. Shuman, Sean Crosson

**Affiliations:** 1 Howard Taylor Ricketts Laboratory, University of Chicago, Argonne National Laboratory, Lemont, Illinois, United States of America; 2 Department of Biochemistry and Molecular Biology, University of Chicago, Chicago, Illinois, United States of America; 3 Department of Microbiology, University of Chicago, Chicago, Illinois, United States of America

## Abstract

*Brucella abortus* is an intracellular bacterial pathogen and an etiological agent of the zoonotic disease known as brucellosis. Brucellosis can be challenging to treat with conventional antibiotic therapies and, in some cases, may develop into a debilitating and life-threatening chronic illness. We used multiple independent assays of *in vitro* metabolism and intracellular replication to screen a library of 480 known bioactive compounds for novel *B. abortus* anti-infectives. Eighteen non-cytotoxic compounds specifically inhibited *B. abortus* replication in the intracellular niche, which suggests these molecules function by targeting host cell processes. Twenty-six compounds inhibited *B. abortus* metabolism in axenic culture, thirteen of which are non-cytotoxic to human host cells and attenuate *B. abortus* replication in the intracellular niche. The most potent non-cytotoxic inhibitors of intracellular replication reduce *B. abortus* metabolism in axenic culture and perturb features of mammalian cellular biology including mitochondrial function and receptor tyrosine kinase signaling. The efficacy of these molecules as inhibitors of *B. abortus* replication in the intracellular niche suggests “dual-target” compounds that coordinately perturb host and pathogen are promising candidates for development of improved therapeutics for intracellular infections.

Approximately 500,000 new cases of human brucellosis are reported annually^1^. However, this number does not fully reflect the total number of cases globally, as the disease remains undiagnosed or misdiagnosed in many areas of Asia, Africa, and South America where it inflicts a significant health, economic, and social burden^2^,^3^. As intracellular pathogens, *Brucella* spp. stably inhabit phagocytes and other host cells, which facilitates successful evasion of the host immune response and has the additional consequence of buffering the cells against antimicrobial compounds. Patients infected with *Brucella* require a one- to three-month course of treatment involving multiple antimicrobial agents^4^,^5^. Even after this extended treatment, the reported incidence of relapse ranges from 3–40%, depending on the course of therapy^6^. The prolonged treatment regimens required to clear *Brucella* infection often have adverse side effects in the patient including hepatotoxicity and gastric damage^5^.

Given that there is no approved human vaccine for brucellosis and that current antimicrobial treatments are long and often harmful to patients, the development of improved treatment strategies for this disease is a high priority. The initial goal of this study was to identify sets of small molecules that either *a)* directly or indirectly inhibit entry and/or replication of *B. abortus* in human macrophages by targeting host factors or *b)* directly inhibit metabolic activity of *Brucella abortus* in defined culture medium. Our host-targeted screen of 480 bioactive molecules from the ICCB chemical library identified 18 molecules that specifically inhibited *B. abortus* replication in the intracellular niche of a human cell line. We further identified 26 pathogen-targeting compounds that inhibited *B. abortus* metabolic activity in axenic culture. The most potent inhibitors of *B. abortus* in the intracellular niche inhibit *B. abortus* in axenic culture and have documented activities against host cells. We conclude that coordinate targeting of host and pathogen pathways may improve the efficacy of treatment of brucellosis and other intracellular bacterial infections.

## Methods

### Bacterial strains

All studies on live *B. abortus* strain 2308 were conducted at Biosafety Level 3 (BSL3) at the University of Chicago, Howard Taylor Ricketts Regional Biocontainment Laboratory according to US Federal Select Agent Program guidelines. mCherry *B. abortus* was previously generated from the wild-type *B. abortus* 2308 parent strain by integration of miniTn7 expressing a single copy mCherry at the *glmS* locus^7^.

### Identification of ICCB compounds that inhibit *B. abortus* metabolism in axenic culture

Prior to compound screening, *B. abortus* 2308 was streaked out and cultivated at 37 °C and 5% CO_2_ for 48 hours on Schaedler blood agar (SBA) plates, re-streaked, and grown for another 48 hours. Cells were scraped off plates and suspended in 1X IF10b medium (Biolog). Cell concentration was adjusted to 5% transmittance at OD_600_ in 1X IF10b medium and diluted by a factor of 1:6.8 in PM9 inoculating solution: 2 mM MgCl_2_•6H_2_O, 1 mM CaCl_2_•2H_2_O, 0.005% yeast extract, 0.005% Tween 40, 2.5 mM D-glucose, 5 mM sodium pyruvate, 1X Dye Mix G (Biolog). Bacterial cell suspension was distributed to each well across six half-area 96-well plates at 50 μL per well. Drugs from the ICCB BioActives library (Enzo Life Sciences) were diluted 1:150 in PM9 inoculating solution and were mixed with cells at a final volume of 100 μL per well resulting in a final drug dilution of 1:300 and bacterial density equivalent to 65% transmittance. Plates were incubated at 37 °C and 5% CO_2_ for two days prior to measuring cell respiration (by assessing reduction of tetrazolium dye mix G at 630 nm) using a Tecan Infinite 200 PRO microplate reader. Compounds that led to a decrease in cell respiration one standard deviation below the mean were deemed hits.

### Identification of compounds structure similarity

The Tanimoto similarity score of assayed drug compounds was obtained using a Chemical Structure Clustering Tool^8^. A Tanimoto similarity score of more than 0.68 was considered statistically significant (i.e. more than two standard deviations away from average similarity score calculated from 50 million compound pairs^9^).

### Mammalian tissue culture and screen for compounds that inhibit *B. abortus* intracellular growth

THP-1 macrophage-like cells were grown to a maximum density of 1 × 10^6^/mL in complete RPMI-1640 medium, 2 mM glutamine (Gibco), and 10% heat-inactivated fetal bovine serum (HyClone) at 37 °C in a humidified environment with 5% CO_2_. Three days prior to infection, THP-1 cells (100 μL aliquots at 1 × 10^6^ cells/mL) were transferred to 96-well plates (Costar) and differentiated with 40 ng/mL phorbol myristate acetate (PMA). After 3 days of differentiation, the medium bathing the confluent cell monolayers was removed by pipetting and replaced with fresh medium. One day prior to infection, mCherry *B. abortus* 2308 was grown from frozen stocks in *Brucella* broth in a shaking incubator at 37 °C, washed, density quantified, and added at multiplicity of infection (MOI) 100 to wells of PMA-differentiated THP-1 cells containing either ICCB drugs (pre-infection treatment) or no drugs (post-infection treatment). In the case of pre-infection treatment, THP-1 cells were incubated with ICCB compounds for 2 hours prior to infection. Tissue culture plates were centrifuged at 2170 rpm for 10 minutes to promote and synchronize infection and then incubated for 1 hour at 37 °C. After this period, 100 μg/mL gentamicin was added to the wells for 1 hour to kill extracellular but not intracellular *B. abortus*. For pre-infection treatment experiments, culture medium was again removed and replaced with fresh medium containing ICCB compounds and 25 μg/mL gentamicin. For post-infection treatment experiments, culture medium was removed and replaced with medium containing 25 μg/mL gentamicin; ICCB compounds were added 4 hours later. Cells were incubated in the presence of ICCB compounds for 3 days before measuring mCherry *B. abortus* fluorescence using a Tecan Infinite 200 PRO microplate reader. Compounds that decreased intracellular fluorescence by ≥20% as normalized to dimethyl sulfoxide (DMSO)-treated control were considered hits.

### Cytotoxicity assays of test compounds against mammalian cells

Two assays were used to assess cytotoxicity: MTT assay and enumeration of host cell nuclei. Reduction of MTT (3-(4,5-dimethylthiazol-2-yl)-2,5-diphenyltetrazolium bromide) was used to measure mammalian (THP-1) cell viability in the presence of all assayed compounds at the end of a three-day incubation period with each of the compounds. MTT was added to cells at a final concentration of 0.5 mg/ml and the cells incubated for 4 hours at 37 °C in a humidified incubator at 5% CO_2_. The medium was replaced with 100 μL isopropyl alcohol containing 40 mM HCl and 20 μL of 10% sodium dodecyl sulfate to solubilize precipitated serum protein. Absorbance of the reaction product was measured at 570 nm in a Tecan Infinite 200 PRO microplate reader. Compounds that decreased reduction of tetrazolium dye by ≥20% of control in an average of two independent experiments were deemed cytotoxic. As a second cytotoxicity assay, we used the established method of nuclei counts per field of view. Briefly, we measured the number of Hoechst-stained nuclei per image in Cell Profiler (v2.1.1) and expressed the nuclei number as an average count from ≈7 images per treatment ± standard deviation from a single experiment.

### Evaluation of morphological changes of THP-1 cells

To determine the effect of compounds on cell morphology, THP-1 cells were treated with compounds as described above and morphological changes were assessed by phase contrast imaging. Compounds deemed toxic induced morphological changes to THP-1 cells identified as cell rounding and shrinkage (Supplementary Fig. S1).

### Quantification of *B. abortus* colony forming units

The effect of drug treatment on *B. abortus* intracellular replication, as assessed by enumeration of colony forming units (CFU), was assayed as follows. 3 days prior to experiment, THP-1 monocytic cells were seeded at 5 × 10^4^ cells per well in a 96-well plate in the presence of 40 ng/mL PMA. One day prior to experiment, *B. abortus* 2308 was grown from frozen stocks in *Brucella* broth in a shaking incubator at 37 °C, washed, density quantified, and added at MOI 100 to wells of differentiated THP-1 cells that were treated with compounds 2 hours prior to, but not during infection. The plates were centrifuged at 2170 rpm for 10 minutes to promote and synchronize infection and then incubated for 1 hour at 37 °C. At that time, 100 μg/ml gentamicin was added for additional hour to kill extracellular, but not intracellular bacteria. After the incubation, medium was removed and replaced with fresh ICCB compounds and 25 μg/mL gentamicin. Cells were incubated in the presence of test compounds for 3 days. After incubation medium was removed and replaced with 200 μL of 0.1% Triton X-100/PBS. The cells were incubated for 10 minutes, mixed by pipetting, and serially diluted in PBS. 10 μL of each serial dilution was spotted on tryptic soy agar plates and incubated for 4 days prior to CFU counting. The results are expressed as an average of 3 to 7 biological replicates.

### Imaging *Brucella*-infected THP-1 cells

THP-1 cells were seeded in a 96-well plate (Greiner), prepared, treated with compounds, and infected with *B. abortus* as described above. After incubation with final candidate compounds, cells were washed three times with 1X PBS and fixed by two 10-minute incubations with 4% paraformaldehyde followed by three washes with 1X PBS. Cell nuclei were labeled with 1 μg/mL Hoechst stain. Cells were imaged using a Leica-DMI6000B fluorescence microscope equipped with a 20X/0.4 NA objective. 6–24 images were captured per each condition using Leica Application Suite X and Hamamatsu Orca-R2 camera. THP-1 nuclei were enumerated to assess compound cytotoxicity in the context of *B. abortus* infection. As described above, Cell Profiler (v2.1.1) was used to measure the number of Hoechst-stained nuclei per image; the number of nuclei was expressed as an average count from ≈7 images per treatment ± standard deviation. The number of intracellular fluorescent *brucellae* was enumerated using the same approach. The effect of the compounds on *B. abortus* intracellular growth was assessed by calculating the number of bacteria per cell represented as a ratio of the total number of bacteria to the total number of nuclei (*brucellae* per nucleus) per image calculated as an average from ≈7 images per treatment ± standard deviation.

### Determination of minimum inhibitory concentration (MIC)

All compounds used to determine MICs were obtained from Santa Cruz Biotechnology except 1400W, which was obtained from Cayman Chemicals. Stock solutions for compounds were prepared by reconstitution in DMSO except for 1400W, which was reconstituted in water. All compound solutions were prepared fresh prior to experiments. MICs of selected compounds were determined by pre-treating cells 2 hours prior to infection. After a 2-hour pre-treatment, cells were infected with *B. abortus* at 100 MOI, cultured for 3 days in the presence of compounds, fixed, stained, and imaged as described above. The number of intracellular bacteria were quantified relative to the number of nuclei using Cell Profiler. MICs represent concentrations of compounds that significantly inhibited number of intracellular *B. abortus* (p < 0.05) at that specific concentration in at least two of four independent experiments. Statistical significance of MICs was determined from an average of ten images from each of the four biological replicates using one-way ANOVA followed by Dunnett’s test.

## Results

### Identification of compounds that inhibit intracellular growth and replication of *Brucella abortus*

**Figure 1:**
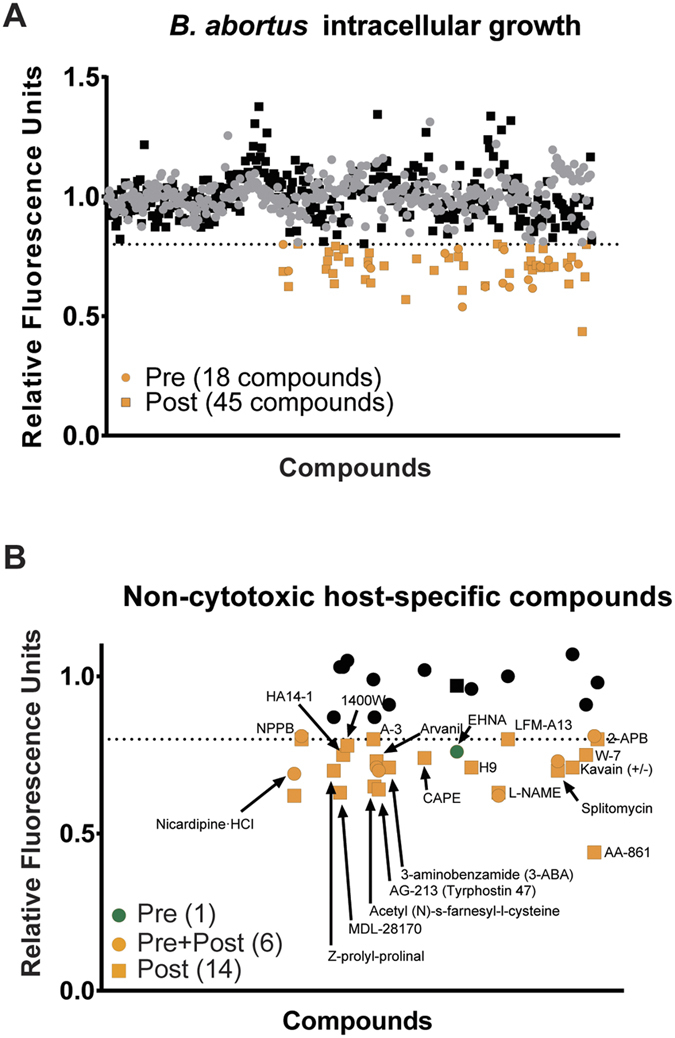
Identification of compounds that inhibit intracellular growth of *B. abortus* in a bulk human cell infection assay. Compounds from the ICCB bioactives library were arrayed into plates containing THP-1 macrophage-like cells infected with wild-type *B. abortus* constitutively expressing mCherry. ICCB compounds are plotted sequentially along the X-axis; relative mCherry *B. abortus* fluorescence is plotted on the Y-axis in all graphs. Dotted line delineates 20% below the mean normalized fluorescence across all conditions; compounds that fall below this line were considered hits (orange). **(A**) Graph of *B. abortus* fluorescence (72 hours post-infection) in the presence of each ICCB compound in cells treated pre-infection (circles) or post-infection (squares) as described in Methods. **(B**) Twenty-one non-cytotoxic compounds that inhibit intracellular growth of *B. abortus* presumably by targeting the host. Orange-filled circles represent compounds that inhibited *B. abortus* infection by 20% or more. Green-filled circle represents a single compound that in our screen was found to be effective only when applied pre-infection.

We sought to identify compounds from the ICCB library of 480 “known bioactives” that target host and/or pathogen to inhibit *B. abortus* infection. This library contains small-molecules whose mechanisms of actions have been established at a molecular level^10^. To quantify growth of *B. abortus* in the intracellular niche, we used a *B. abortus* strain^7^ that constitutively expresses mCherry fluorescent protein from a Tn7 transposon integrated in single copy at the chromosomal Tn7 attachment site (*glmS*)^11^. We screened for compounds that prevent entry (i.e., are effective when host cells are treated before infection, but not after infection) or intracellular replication. Specifically, we treated PMA-differentiated THP-1 macrophage-like cells with compounds from the ICCB library either 2 hours prior to infection or 4 hours after infection. The infected cells were incubated for an additional 72 hours prior to measuring fluorescent signal from intracellular *B. abortus*-mCherry. Using an MTT assay^12^, we assessed compound cytotoxicity to THP-1 cells at 72 hours (Supplementary Table S1). Compounds that decreased intracellular *Brucella* fluorescence by 20 percent or more in an initial bulk fluorescence screen were considered hits. This screen yielded 45 compounds that inhibited intracellular *B. abortus* growth when applied post infection, 20 of which were non-cytotoxic (Fig. 1). The pre-infection screen yielded 18 compounds that inhibited intracellular growth, seven of which were non-cytotoxic (Fig. 1). Six of the seven non-cytotoxic compounds identified in the pre-infection screen were among the 20 that inhibited growth when applied after infection (Fig. 1B). A single compound, EHNA, was effective only when applied prior to infection, suggesting that it may prevent *Brucella* entry into the host cell. In total, we identified 21 non-cytotoxic compounds that inhibit *B. abortus* infection/intracellular replication based on a bulk fluorescence assay (Supplementary Table S2). Validation of the hits identified using this fluorescence-based screen by two additional methods, enumeration of CFU and microscopy, is described below.

### Identification of compounds that inhibit metabolic activity of *Brucella abortus*

**Figure 2:**
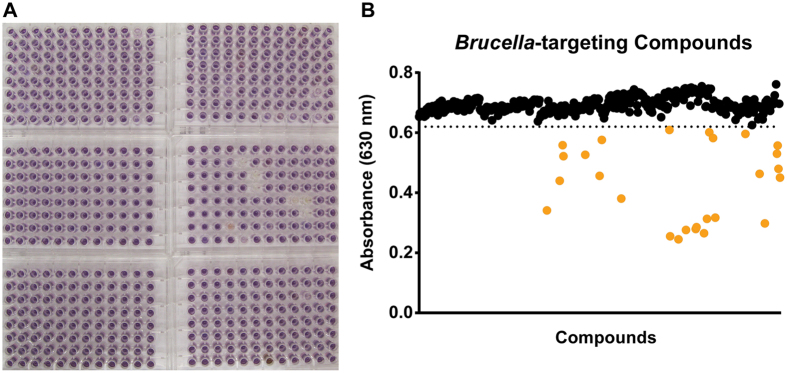
Screen for molecules that inhibit *Brucella abortus* metabolism *in vitro*. (**A**) A panel of six 96-well plates arrayed with 480 small molecules from the ICCB bioactives library. *B. abortus* growth/metabolism was assessed colorimetrically in test and control wells containing a minimal-defined base medium and tetrazolium dye as a metabolic indicator (purple color, monitored at 630 nm) as described in Methods. (**B**) *B. abortus* metabolism in minimal-defined medium in the presence of 480 ICCB compounds, as assessed by quantifying tetrazolium formazan absorbance at 630 nm. Orange circles represent twenty-six compounds that inhibited *Brucella* metabolism by ≥1 standard deviation from the mean (dotted horizontal line represents 1 standard deviation below the mean). ICCB compounds are plotted sequentially along the x-axis. The twenty-six hit compounds are listed in Table 1.

We next sought to identify compounds that directly inhibited *B. abortus* metabolism in axenic culture. To accomplish this, we adopted a modified phenotype microarray platform to screen the ICCB library for compounds that decrease metabolic activity of *B. abortus* cells grown in the presence of each compound over a period of 2 days. We monitored the metabolic activity of *B. abortus* by measuring reduction of tetrazolium dye spectrophotometrically: tetrazolium is reduced by metabolically active cells, eliciting a color change. This screen identified molecules that inhibited metabolic activity in a defined liquid medium growth platform^13^. Of the 480 ICCB compounds, we identified 26 that decrease *B. abortus* metabolic activity by ≥1 standard deviation from the mean (Fig. 2, Table 1). Three of these 26 *Brucella*-targeting compounds (Table 1) overlapped with the 21 compounds that inhibit intracellular *B. abortus* (Supplementary Table S2). We thus refer to the remaining 18 of the 21 compounds that inhibit intracellular *Brucella* infection/replication as host-specific compounds. We note that the 26 *Brucella*-targeting compounds reported here are not clinical antibiotics. The molecular targets of these compounds have been established in eukaryotic cells in some cases, though mechanisms of action may differ against bacteria.

### Compounds with activities against host and pathogen potently inhibit intracellular replication of *B. abortus*

**Figure 3:**
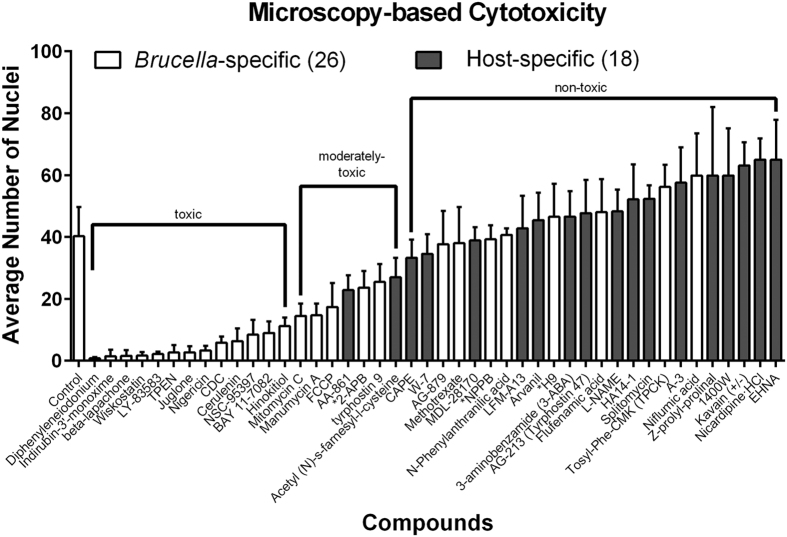
Microscopy-based assessment of hit compound cytotoxicity in *Brucella*-infected THP-1 cells. Cytotoxicity of the 26 *Brucella*-specific (white bars) and 18 host-specific compounds (shaded bars) was assessed by quantifying the average number of nuclei in a viewing area per each treatment. The results are expressed as an average number of nuclei calculated from 6–24 images per treatment in a single experiment. Error bars represent standard deviation. Toxic compounds were also identified based on a qualitative image analysis. Compounds that had a significantly lower average number of nuclei than control (p < 0.05) were deemed toxic. While compounds that are labeled as moderately-toxic had a lower average number of nuclei, the image analysis revealed adherent cells that had normal morphology (Supplementary Fig. S1). Compounds that had no apparent affect on morphology and had a high average number of nuclei were deemed non-toxic. *Three overlapping host- and *Brucella*-specific compounds.

We next sought to validate the cytotoxicity and inhibitory efficacy of hits presented above using additional experimental approaches. As described above, compound cytotoxicity to THP-1 cells was initially assessed using the MTT assay, which measures mitochondrial function. To avoid identification of false-positive cytotoxic compounds, we assayed cytotoxicity using two additional approaches. Specifically, we tested whether the 26 pathogen-targeting compounds (Table 1) and 18 host-specific compounds (Supplementary Table S2) were cytotoxic to THP-1 cells infected with *B. abortus* by imaging infected cells by fluorescence microscopy, (***i***) assessing defects in host cell morphology, and (***ii***) quantifying the average number of host nuclei per imaged field upon drug treatment (Fig. 3). Both of these are established methods to assess cell viability^14^,^15^. Of the 44 total compounds, 13 were toxic to human cells infected with *B. abortus* based on these imaging assays. All 13 of the cytotoxic compounds were identified as inhibitors of *B. abortus* metabolism in axenic culture (Table 1). Toxicity of 12 of the 13 compounds was consistent with the MTT cytotoxicity assay (Supplementary Table S1), which was measured on uninfected THP-1 cells. We removed these 13 compounds from further analysis. We retained seven compounds that were moderately cytotoxic based on quantification of host nuclei, but which did not result in noticeable defects in host cell morphology (Supplementary Fig. S1, Supplementary Table S3).

**Figure 4:**
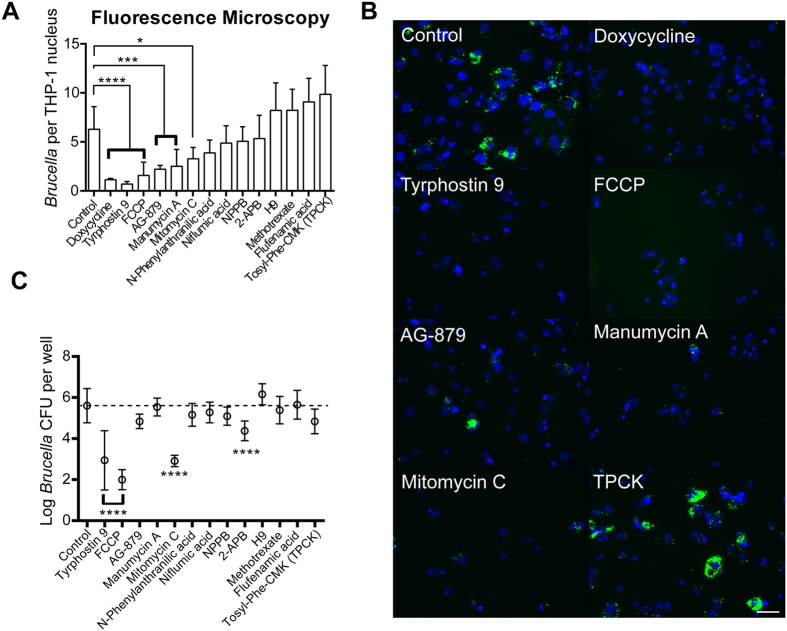
Efficacy of non-cytotoxic pathogen-targeting compounds as inhibitors of *B. abortus* intracellular replication in a THP-1 infection model. (**A**) Quantification of inhibitory efficacy of compounds, measuring the average ratio of bacteria-to-nucleus in THP-1 cells treated with each of the 13 non-cytotoxic pathogen-targeting compounds in a single experiment. Statistical significance was evaluated from ≈7 images per treatment using one-way ANOVA followed by Dunnett’s test (*p < 0.05, ***p < 0.001, ****p < 0.0001). Error bars represent +/− standard deviation. (**B**) Sample fluorescence images of nuclei (blue) and intracellular *B. abortus* (pseudocolor green) assessing the efficacy of each of the 5 final pathogen-targeting compounds that significantly inhibited infection by *B. abortus*: tyrphostin 9, FCCP, AG-879, manumycin A, and mitomycin C. Doxycycline is a positive control and TPCK represents a non-hit compound that did not have any effect on infection. Scale bar = 50 μm. (**C**) *B. abortus* CFU counts generated from infected THP-1 treated with 13 identified non-toxic compounds that inhibit *B. abortus* metabolism in axenic culture. Data represent an average from 3–7 biological replicates. Error bars represent +/− SEM.

To test whether compounds identified as inhibitors of *B. abortus* metabolism in axenic culture also inhibited replication in the intracellular niche, we imaged infected THP-1 cells and quantified fluorescent brucellae. Specifically, we calculated the average bacteria-to-host nucleus ratio in the presence of each of the 13 *Brucella*-targeting compounds that were deemed either non-cytotoxic or moderately cytotoxic to THP-1 (Fig. 3, Supplementary Table S3). This imaging approach identified five compounds that significantly inhibited intracellular replication of *B. abortus* (Fig. 4A,B). As further validation, we lysed THP-1 cells treated with this same set of hit compounds and plated for *B. abortus* CFUs 3 days post-infection. Enumeration of *B. abortus* CFUs from infected THP-1 cells confirmed the efficacy of 3 out of the 5 compounds as inhibitors of *B. abortus* replication and survival in the intracellular niche; treatment of THP-1 with 2-APB showed significant reduction of CFU, but no significant difference in our imaging assay (Fig. 4C). Only three hits, tyrphostin 9, carbonyl cyanide-4-(trifluoromethoxy)phenylhydrazone (FCCP), and mitomycin C, were significantly effective (p-value < 0.05) across all three functional assays. N-phenylanthranilic acid, niflumic acid, NPPB, 2-APB, H9, methotrexate, flufenamic acid, AG-879, manumycin A, and TPCK did not inhibit *B. abortus* infection in all three assays at the tested concentrations but, given different sensitivities of each assay and the ability of these compounds to inhibit *B. abortus* metabolism in axenic culture, these molecules still represent candidate inhibitors of *Brucella* in the intracellular niche. Our final list of hits includes 13 compounds that were initially identified as metabolic inhibitors. Of these 13 hits, three robustly inhibited intracellular *B. abortus* replication across all three assays, three compounds were effective in two assays. Seven hit compounds inhibited only in the initial screen (Table 2).

**Figure 5:**
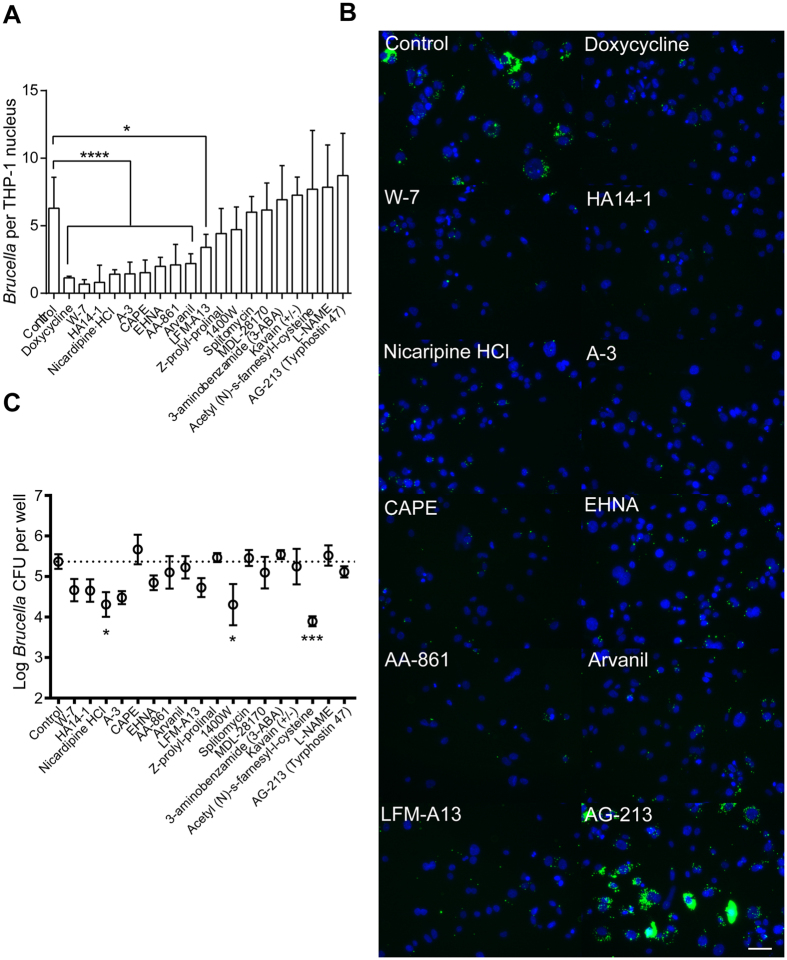
Efficacy of non-cytotoxic host-specific compounds as inhibitors of *B. abortus* intracellular replication in a THP-1 infection model. (**A**) Quantification of inhibitory efficacy of compounds, measuring the average ratio of bacteria-to-nucleus in THP-1 cells treated with each of the 18 non-cytotoxic host-specific compounds in a single experiment. Statistical significance was evaluated from ≈7 images per treatment using one-way ANOVA followed by Dunnett’s test (*p < 0.05, ****p < 0.0001). Error bars represent +/− standard deviation. (**B**) Sample fluorescence images of nuclei (blue) and intracellular *B. abortus* (pseudocolor green) assessing efficacy of each of the 9 final host-specific compounds that significantly inhibited infection by *B. abortus.* Doxycycline is a positive control and AG-213 represents a non-hit compound that did not have any effect on infection. Scale bar = 50 μm. (**C**) *B. abortus* CFU counts generated from infected THP-1 treated with 18 identified non-toxic compounds that presumably target host cells to inhibit *B. abortus* infection. Data represent an average from 3–7 biological replicates. Error bars represent +/− SEM.

We applied the same image analysis and CFU-enumeration pipeline to test the efficacy of the 18 non-cytotoxic host-specific compounds (Fig. 3) that had no effect on *B. abortus* metabolism in axenic culture (Supplementary Table S3). This approach provided us with independent efficacy measurements of compounds that inhibit replication of intracellular *Brucella* in the initial bulk fluorescence assay. Imaging showed that 9 of 18 non-cytotoxic compounds identified in the bulk fluorescence screen significantly inhibited replication of intracellular bacteria (Fig. 5A,B, Table 2). The CFU assay confirmed a single hit, nicardipine, which was also effective in two previous functional assays (Fig. 5C). Hence, one of the 18 non-cytotoxic host-specific compounds showed efficacy across all three inhibition assays and nine were effective in two assays. The remaining eight hits showed efficacy only in the initial screen (Table 2).

**Figure 6:**
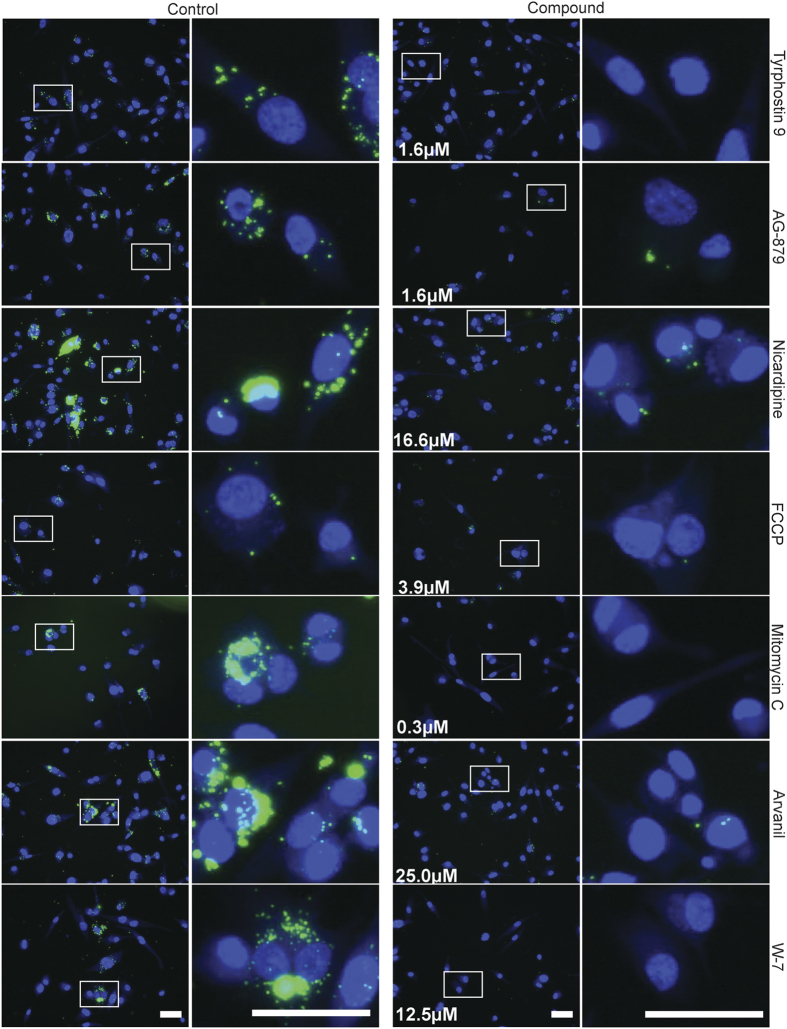
Inhibition of *B. abortus* replication in THP-1 cells at MIC. Images of *B. abortus* (green pseudocolor) and Hoechst-stained THP-1 nuclei (blue) of cells treated with compunds at their respective minimum inhibitory concentrations (MICs). The images were taken 72 hours post-infection. Control column represents mock treated controls matching each treatment in the Compound column. Magnified inset regions (white box) appear to the right of each image. Scale bars = 50 μm.

Each of the functional assays used to identify anti-infective activity of compounds offers different sensitivity. To more closely examine inhibitory efficacy, we determined the minimum inhibitory concentrations (MICs) of selected compounds that were effective across at least two functional assays; these included tryphostin 9, AG-879, nicardipine, FCCP, mitomycin C, arvanil, W-7, 2-APB, and 1400W. The MICs were determined based on the lowest compound concentration that significantly (p < 0.05) decreased count of intracellular bacteria as assessed by fluorescence microscopy (Supplementary Fig. S4). While the MICs for seven compounds were lower than the initial screen concentration, two compounds, 2-APB and 1400W, did not show significant inhibition at concentrations lower than the initial screen (Table 3; Fig. 6). These results are consistent with our initial imaging data presented in Table 2 and Figs 4 and 5. The MIC measurements support the conclusion that these compounds are inhibitors of *B. abortus* in the intracellular niche

In summary, we have identified 4 robust *B. abortus* antimicrobials, one of which (nicardipine) inhibits replication specifically in the host niche but not in axenic culture. Three compounds: tyrphostin 9, FCCP, and mitomycin C, have well-established molecular targets in the host-cell, but also inhibit *Brucella* metabolism and its intracellular replication (Table 2). The latter category of compounds, which have activities against host and pathogen, are the most potent inhibitors in our screen (Figs 4C and 5C, Tables 2 and 3). These compounds represent a potentially novel class of *Brucella* anti-infectives; their efficacy may arise from their ability to coordinately perturb host and pathogen. The remaining host- and pathogen-targeting compounds (Table 2) represent promising anti-infective hits that may be developed for combination therapeutic approaches that simultaneously target pathogen and host.

## Discussion

### Activities and Targets of Compounds that inhibit *Brucella* metabolism in axenic culture

We identified 26 compounds that directly target *B. abortus* to inhibit its metabolic activity (Table 1). The largest class of inhibitory compounds is kinase inhibitors. While the mechanism(s) by which these compounds inhibit *B. abortus* respiration remain undefined, previous reports have demonstrated that eukaryotic kinase inhibitors have antibacterial activity^7^,^16^,^17^. Of these 26 compounds, 5 exhibit significant structural similarity: flufenamic acid, 5-Nitro-2-(3-phenylpropylaminol)benzoic acid (NPPB), n-phenylanthranilic acid, niflumic acid, and LY-83583 (Supplementary Fig. S2A). All, with the exception of LY-83583, are known to target ion homeostasis, which is the second largest class of compounds that inhibit *B. abortus* metabolism in axenic culture (Table 1). Two naphthoquinones, juglone and beta-lapachone, also exhibit significant structural similarity (Supplementary Fig. S2A). Although each of these compounds are potent modulators of eukaryotic cell biology, both have reported antibacterial activity^18^,^19^ suggesting they may coordinately perturb host and pathogen. Four additional classes of hit compounds include metal chelators, inhibitors of energy metabolism, DNA modifiers, and inhibitors of lipid biosynthesis. The identification of metal chelators as inhibitors is not surprising given that a number of metals, including zinc, iron, manganese, and magnesium are essential for *B. abortus* growth in *in vivo* and *in vitro* models^20^,^21^. Nonetheless, they provide attractive therapeutic candidates.

Three *Brucella*-targeting compounds, FCCP, diphenyleneiodonium, and methotrexate, are known to target energy metabolism and may therefore intercept *Brucella* metabolic pathways and decrease the cellular pool of reducing equivalents. Among these three compounds, methotrexate, is an FDA-approved antimetabolite drug known for inhibiting dihydrofolate reductase (DHFR). Interestingly, FDA-approved DHFR inhibitors are used as antiprotozoal and antimicrobials^22^. FCCP, a known uncoupling ionophore was previously reported to inhibit intracellular transport of secretory viral glycoproteins at concentrations overlapping with the MIC identified in our study^23^. Two DNA-targeting agents, mitomycin C and beta-lapachone, are also established anti-infectives^24^,^25^. Both cerulenin and cinnamyl-3,4-dihydroxy-α-cyanocinnamate (CDC) are known inhibitors of lipid^26^,^27^ and sterol biogenesis^28^ in eukaryotes, but also exhibit a direct antimicrobial activity^29^,^30^. Sterols have been reported as important host factors in macrophage infection by *B. abortus*^31^. CDC, in particular, is notable as it inhibits 12- and 5-lipoxygenase^27^, which have specific host activities that are required for *Streptococcus pneumoniae* lung infection and *B. abortus* infection in mice^32^,^33^. Cerulenin and CDC thus have the potential to target both the host and the pathogen in certain infections. The remaining five compounds fall into the “Other” class, these include: NSC-95397, LY-83583, wiskostatin, nigericin, and tosyl-phe-CMK (TPCK). Previous studies report antibacterial activities of wiskostatin and nigericin^34^,^35^. Collectively, many of these 26 compounds represent novel metabolic inhibitors that effectively target *Brucella* and perhaps other bacteria.

### Pharmacological Features of *Brucella*-targeting compounds that inhibit replication in the intracellular niche

Though each of the 26 identified compounds shown in Table 1 potently inhibit bacterial metabolism, many are cytotoxic to human cells at our screen concentrations. Thirteen of the 26 *Brucella*-targeting compounds were classified as non-toxic or moderately toxic to THP-1 cells based on our assays (Fig. 3). Of the 13 compounds, tyrphostin 9, FCCP, and mitomycin C, were robust inhibitors of *B. abortus* replication in the intracellular niche across all three inhibition assays (Table 2). An additional three *Brucella*-targeting compounds, AG-879, manumycin A, and 2-APB, were effective in two of the three intracellular replication assays. The remaining seven hits effectively inhibited intracellular replication as measured the initial fluorescence screen, but inhibition was not observed by fluorescence imaging and enumeration of CFUs (Table 2).

Many non-cytotoxic compounds that inhibit *B. abortus* in pure culture have well-documented ability to perturb eukaryotic cellular processes. Notably, a number of compounds that inhibit *B. abortus* in axenic medium, including tyrphostin 9, AG-879, FCCP, manumycin A, and mitomycin C, are known to affect mitochondrial function either by targeting the oxidative phosphorylation or mitochondrial membrane potential^36^,^37^,^38^,^39^. In addition to overlapping function in targeting mitochondria, tyrphostin 9 and AG-879 have almost identical structures and have also been reported to inhibit protein kinases (Supplementary Fig. S3)^40^,^41^. Host-kinase signaling has been implicated in a number of viral and bacterial infections, including infection by *B. abortus*^42^,^43^,^44^,^45^,^46^. FCCP is also reported to disrupt host microtubules, which is essential for *Brucella* infection^46^,^47^. Manumycin A, a compound that inhibits *B. abortus* metabolism, is a known inhibitor of farnesyltransferase activity in eukaryotic cells^48^, and has been reported to inhibit *Anaplasma phagocytophilum* by directly targeting the pathogen, but not the host^49^.

Although, it is not known whether these compounds inhibit *B. abortus* in the intracellular niche by targeting the pathogen only, or by targeting both the host and *B. abortus*, we note interesting congruence between our final hit list with compounds that target host pathways to inhibit Type IV effector protein translocation by *Legionella pneumophila*^50^. Tyrphostin 9, W-7, caffeic acid phenethyl ester (CAPE), FCCP, and HA14-1 are among the overlapping *Legionella/Brucella* hits, and Type IV secretion is a crucial requirement of *B. abortus* virulence in cell-based and animal infection models^51^,^52^. The coordinate effects of these compounds on host and microbial energy metabolism and host cell pathways may contribute to their efficacy as inhibitors of intracellular *B. abortus* replication.

### Activities and Targets of Host-specific compounds that inhibit replication of *B. abortus* in the intracellular niche

Eighteen of the thirty-one compounds that inhibited replication of intracellular *B. abortus* had no direct effect on *Brucella* metabolism in axenic culture. Therefore, these are candidate compounds that inhibit intracellular *B. abortus* replication by targeting the host, but not the pathogen. The inhibitory efficacy of a single compound, nicardipine, was consistent across all three assays (fluorescence, cell imaging, and CFU counts), nine compounds were effective across two assays, and the remaining eight inhibited infection only in the initial fluorescence screen (Table 2).

Two of the five host-specific compounds that target host kinase signaling pathways effectively inhibited infection across two functional assays. These include A-3 and W-7, which is a calmodulin antagonist (Table 2)^53^. A-3 and W-7 are structurally similar (Supplementary Fig. S2B, Supplementary Fig. S3), suggesting that they may target the same host pathway to interfere with intracellular *Brucella* growth and replication; the cAMP/cGMP-dependent protein kinases, PKA and PKG, are common targets of these compounds^54^,^55^. Erythro-9-(2-hydroxy-3-nonyl)adenine (EHNA) is an inhibitor of phosphodiesterase-2 (PDE2) that hydrolyzes cAMP and cGMP, which are responsible for activation of PKA and PKG, respectively^56^,^57^. Among all host-targeting compounds, two pairs showed significant structure similarity: ion channel ligands (nicardipine and NPPB) and protease inhibitors (MDL-28170 and Z-prolyl-prolinal) (Supplementary Fig. S2B). Structural similarity between these compounds suggests shared anti-infective mechanisms of action, though follow-up experiments focused on mechanism of action are required.

Four additional hits, W-7, nicardipine, arvanil, and kavain, are known to affect calcium homeostasis. W-7 was previously reported to have antibacterial activity against a number of intracellular bacterial pathogens that can replicate inside host cells, including *Ehrlichia* and *Pseudomonas*^58^,^59^. Our previous work on host-targeted anti-infectives identified a number of calcium channel-targeting FDA-approved drugs that were effective in the inhibition of intracellular bacteria^7^. Nifedipine, a dihydropyridine calcium channel blocker that is structurally related to nicardipine, was shown to inhibit *Brucella* infection in bone marrow-derived macrophages. This result is congruent with the data reported herein, and provides evidence that these compounds will be effective in different cell types^60^. Together, these results further underscore the importance of host calcium homeostasis during *Brucella* intracellular infection and growth.

AA-861, HA14-1, and CAPE, had no effect on *B. abortus* growth and replication in axenic culture, but inhibited intracellular *B. abortus* replication across two functional assays. In agreement with our finding that AA-861, a 5-lipoxygenase (5-LO) inhibitor, attenuates intracellular growth and replication of *Brucella*, a recent study demonstrated that 5-LO deficient mice are resistant to infection by *B. abortus*^33^. Notably, CAPE, an NF-κB inhibitor, was also shown to inhibit 5-LO^61^. Identification of HA14-1, a pro-apoptotic Bcl-2 inhibitor, suggests that activating apoptosis may interfere with *Brucella* infection. Indeed, inhibition of apoptosis is reported to be beneficial to *Brucella* intracellular growth and survival^62^,^63^. Finally, of the two nitric oxide synthase (NOS) inhibitors, 1400W and L-NAME, only 1400W was effective across two growth inhibition assays. Identification of these inhibitors suggests that nitric oxide may be beneficial to the growth/replication of *B. abortus* in THP-1 macrophages. Infection of mammalian cells by *Listeria monocytogenes* relies on host-derived nitric oxide generated by NOS^64^. However, inhibition of murine NOS was shown to promote *Brucella* infection, but only under specific conditions (using IFN-γ-treated cells and opsonized-*brucellae*)^65^. The exact mechanism of action of each of the newly identified *B. abortus* inhibitors remains to be determined.

In summary, we identified molecules that inhibit *B. abortus* cellular metabolism and intracellular growth. Our data suggest that molecules targeting both pathogen and host can enhance inhibition of intracellular *B. abortus* replication and survival in a human macrophage infection model. Among the identified inhibitors of *B. abortus* in the intracellular niche, we note enrichment of broad-spectrum kinase inhibitors, compounds that target ion homeostasis, and compounds that target mitochondrial function. Compounds that target host and pathogen, either as a single “dual-target” molecules or in combination, may be developed for use against a broader spectrum of intracellular microbial infections, including eukaryotic parasites. Like other host-targeting molecules, dual-targeting compounds hold the potential of limiting the emergence of antibiotic resistance^66^. Future studies in animal models of infection and disease will test the antimicrobial efficacy of these compounds *in vivo*.

## Additional Information

**How to cite this article**: Czyż, D. M. *et al.* A dual-targeting approach to inhibit *Brucella abortus* replication in human cells. *Sci. Rep.* **6**, 35835; doi: 10.1038/srep35835 (2016).

## Supplementary Material

Supplementary Information

## Figures and Tables

**Table 1:** ICCB compounds that inhibit *Brucella abortus* metabolic activity in minimal-defined medium.

Compound	Description	CID	% MA
Posttranslational modifications			
Tyrphostin 9	PDGF-R tyrosine kinase inhibitor	5614	43
AG-879	Tyrosine kinase inhibitor	5487525	55
H9	Kinase inhibitor	11957465	40
BAY 11–7082	IKK kinase inhibitor	5353431	38
Indirubin-3′-monoxime	GSK-3beta kinase inhibitor	5326739	86
Juglone	PIN1 inhibitor	3806	41
Manumycin A	Ras farnesylation inhibitor	6438330	87
**Ion homeostasis**			
2-APB	IP3 receptor inhibitor	1598	77
N-Phenylanthranilic acid	Misc. channels	4386	81
Flufenamic acid	Potassium channels	3371	50
NPPB	Misc. channels	4549	76
Niflumic acid	Misc. channels	4488	64
**Metal chelators**			
Hinokitiol	Iron chelator	3611	88
TPEN	Heavy metal chelator	5519	67
**Energy metabolism**			
FCCP	Mitochondrial uncoupler	3330	36
Diphenyleneiodonium	Flavoprotein inhibitor	3101	37
Methotrexate	DHFR inhibitor	126941	84
**DNA modification**			
Mitomycin C	Cross links DNA	5746	46
beta-lapachone	Topoisomerase 1 inhibitor	3885	84
**Lipid biosynthesis**			
Cerulenin	Fatty acid biosynthesis inhibitor	5282054	70
CDC	12-Lipoxygenase inhibitor	9905190	81
**Other**			
NSC-95397	CDC25 phosphatase inhibitor	262093	40
LY-83583	Inhibits NO-activation of guanylate cyclase	3976	45
Wiskostatin	N-WASP inhibitor	2775510	65
Nigericin	Induces intracellular acidification	34230	66
Tosyl-Phe-CMK (TPCK)	Serine protease inhibitor	439647	76

Compounds that inhibit *B. abortus* metabolic activity fall into seven specific classes based on their ICCB library annotations: posttranslational modifications, ion homeostasis, metal chelators, energy metabolism, DNA modification, lipid biosynthesis, and other. Each compound has a corresponding PubChem Compound Identifier (CID) and a corresponding percent metabolic activity (% MA) normalized to wild-type control (expressed as % of control).

**Table 2:** Thirty-one non-toxic host- and pathogen-targeting antimicrobial compounds that inhibit intracellular replication of *B. abortus* in a THP-1 infection model in any of the three inhibition assays.

Compound		CIDs	%MA	Target		Assay		
	Description			THP-1	*Bab*	1	2	3
Kinase signaling								
Tyrphostin 9	PDGF-R tyrosine kinase inhibitor	5614	43		X	+	+	+
AG-879	Tyrosine kinase inhibitor	5487525	55		X	+	+	−
H9	Kinase Inhibitor	11957465	40		X	+	−	−
A-3	Kinase Inhibitor	9861903	102	X		+	+	−
EHNA	PDE2 inhibitor/adenosine deaminase inhibitor	11957547	101	X		+	+	−
AG-213 (Tyrphostin 47)	EGF-R tyrosine kinase inhibitor	5485187	103	X		+	−	−
LFM-A13	BTK inhibitor	9549280	99	X		+	−	−
**Ion homeostasis**								
2-APB	IP3 receptor inhibitor	1598	77		X	+	−	+
N-phenylanthranilic acid	Miscelaneous channels	4386	81		X	+	−	−
Flufenamic acid	Potassium channels	3371	50		X	+	−	−
NPPB	Miscelaneous channels	4549	76		X	+	−	−
Niflumic acid	Miscelaneous channels	4488	64		X	+	−	−
Nicardipine·HCl	Calcium channels	41114	95	X		+	+	+
Arvanil	Vaniloid receptor agonist	6449767	106	X		+	+	−
W-7	Calmodulin antagonist/MLC kinase inhibitor	124887	101	X		+	+	−
Kavain (+/−)	Voltage-dependent Na channel inhibitor	5369129	100	X		+	−	−
**Protease inhibitors**								
Tosyl-Phe-CMK (TPCK)	Serine protease inhibitor	439647	76		X	+	−	−
MDL-28170	Calpain inhibitor	72430	101	X		+	−	−
Z-prolyl-prolinal	Prolyl endopeptidase inhibitor	122623	103	X		+	−	−
**Energy metabolism**								
FCCP	Mitochondria uncoupler	3330	36		X	+	+	+
Methotrexate	DHFR inhibitor	126941	84		X	+	−	−
**Nitric oxide**								
1400W	iNOS inhibitor	2733515	103	X		+	−	+
L-NAME	NOS inhibitor	135193	100	X		+	−	−
**Farnesylation**								
Manumycin A	Ras farnesylation inhibitor	6438330	87		X	+	+	−
Acetyl (N)-farnesyl-l-cysteine	Farnesylation inhibitor	1994	105	X		+	−	+
**Other**								
Mitomycin C	Cross links DNA	5746	46		X	+	+	+
HA14-1	Bcl-2 ligand induces apoptosis	3549	100	X		+	+	−
CAPE	Antioxidant/ NFkappa B inhibitor	5281787	97	X		+	+	−
AA-861	5-lipoxygenase inhibitor	1967	105	X		+	+	−
Splitomycin	Sir2p inhibitor	5269	96	X		+	−	−
3-aminobenzamide (3-ABA)	ADP ribose polymerase, apoptosis inhibitor	1645	103	X		+	−	−

Eighteen host-targeting and 13 pathogen-targeting non-cytotoxic compounds that inhibit intracellular growth of *B. abortus* fall into seven categories. Each compound has a corresponding PubChem Compound Identifier (CID); treatment results in fractional metabolic activity (MA) normalized to wild-type control (expressed as % of control). Compounds that target the host cell (THP-1) or *B. abortus* (*Bab*) metabolic activity directly are marked with X. Each of the compounds is marked either as an effective (+) or non-effective (−) hit across any of the three functional assays (1-bulk intracellular fluorescence screen in 96-well plate format, 2- fluorescence microscopy enumeration of bacteria per nucleus, and 3-enumeration of *B. abortus* CFU isolated from infected THP-1); p-values of less than 0.05 were defined as hits as described in Materials and Methods.

**Table 3:** Minimum Inhibitory Concentrations.

Compound	Screen conc. (μM)	MIC (μM)
Tyrphostin 9	59.0	1.6
AG-879	52.7	1.6
Nicardipine	32.3	16.7
FCCP	65.6	3.9
Mitomycin	49.9	0.3
Arvanil	37.9	25.0
W-7	44.2	12.5
2-APB	74.0	133.0
1400W	94.0	>133.3

MICs of selected compounds that inhibited intracellular replication of *B. abortus* in THP-1 cells in at least two different functional assays. MIC is defined as the compound concentrations that significantly (p < 0.05) decreased the number of intracellular bacteria compared to untreated control in a fluorescence cell imaging assay. Statistical significance of MICs was determined from an average of ten images from each of the four biological replicates (treated versus untreated cells; one-way ANOVA followed by Dunnett’s test).
