# Supplementary material for: A dual-targeting approach to inhibit Brucella abortus replication in human cells
Source: Sci Rep. 2016 Oct 21;6:35835. doi: 10.1038/srep35835 (PMC5073326; doi:10.1038/srep35835)
Supplement: Supplementary Information [file srep35835-s1.pdf]

A dual-targeting approach to inhibit *Brucella abortus* replication in human cells

Daniel M. Czyż, Neeta Jain-Gupta, Howard A. Shuman, and Sean Crosson

Supplementary Materials

Table S1. Assessment of compound toxicity to THP-1 cells.
Table S2. Twenty-one compounds that inhibit infection by *B. abortus.*
Table S3. Microscopy-based assessment of cytotoxicity of compounds that inhibit infection by *B. abortus*.

Figure S1. Qualitative assessment of cell morphology.
Figure S2. Analysis of chemical structures similarities of *Brucella*-targeting compounds.
Figure S3. Compound structures.
Figure S4. Quantification of intracellular *B. abortus* at dose-response.

Supplementary Table S1. Assessment of compound toxicity to THP-1 cells.

149 toxic compounds (more than 20% cytotoxicity)		
Comp. No.	Name	Screen Conc. [μM]
15	13,14-Dihydro-PGE1	3.33
16	13-cis retinoic acid	3.33
23	16,16-Dimethyl-prostaglandin E2	3.33
24	17-Octadecynoic acid	3.33
31	1-Octadecyl-2-methylglycero-3 PC	3.33
48	6-Formylindolo [3,2-B] carbazole	3.33
57	9b,11a-Prostaglandin F2	3.33
58	9-cis Retinoic acid	3.33
64	AM-580	3.33
81	C-PAF	3.33
119	Misoprostol, free acid	3.33
134	Prostaglandin E1	3.33
135	Prostaglandin E2	3.33
140	All trans retinoic acid	3.33
157	Amiodarone·HCl	24.45
158	A-23187	31.83
161	Bepridil·HCl	41.36
165	Dichlorobenzamil·HCl	39.21
171	Flunarizine·2HCl	34.91
172	Fluspirilene	35.05
181	Loperamide·HCl	32.46
186	Nifedipine	48.12
188	Niguldipine	25.79
194	Paxilline	38.27
196	Penitrem A	26.28
197	Phenamil	54.52
200	Pimozide	36.11
203	Propafenone	48.81
208	SDZ-201106	35.72
209	SKF-96365	45.48
210	Tetrandine	26.76
211	Thapsigargin	25.61
224	NapSul-Ile-Trp-CHO	33.90
225	3,4-dichloroisocoumarin	77.51
228	Ala-Ala-Phe-CMK	49.05
230	Ac-Leu-Leu-Nle-CHO	43.46
234	Betulinic acid	36.49
238	Triptolide	46.24
239	Tanshinone IIA	56.62
240	Nigericin	22.99
241	Lycorine	58.01
242	10-hydroxycamptothecin	45.74

Comp. No.	Name	Screen Conc. [µM]
243	beta-lapachone	68.79
244	Parthenolide	67.12
245	MG-132	35.04
246	QNZ	46.76
253	Zardaverine	62.14
263	Actinomycin D	35.88
265	AG1478	47.32
269	AG-879	52.67
270	Alamethicin	8.48
271	AG-1296	62.59
274	Ikarugamycin	34.68
277	17-Allylamino-geldanamycin	28.46
278	Anisomycin	62.82
279	Aphidicolin	49.24
280	Aristolochic acid	48.84
286	BAPTA-AM	21.80
289	IB-MECA	32.66
290	Brefeldin A	59.45
300	Calphostin C	2.11
301	Calyculin A	1.65
302	Camptothecin	47.84
303	Cantharidin	84.95
308	CGP-37157	51.40
309	Chelerythrine	47.84
316	Curcumin	45.24
317	Cycloheximide	59.24
318	Cyclosporin A	13.86
323	Damnacanthal	59.05
324	YC-1	54.76
328	Dexamethasone	42.47
331	GW-9662	60.24
333	Hinokitiol	101.50
334	Diphenyleneiodonium	59.72
336	Doxorubicin	28.74
337	DRB (Benzimidazole)	52.22
343	Estradiol	61.19
344	Etoposide	28.32
345	FCCP	65.57
346	Forskolin	40.60
347	Geldanamycin	29.73
348	Genistein	64.01
349	Ro 31-8220	36.43
351	Go6976	44.04
353	H7	57.20
354	H-89	37.34

Comp. No.	Name	Screen Conc. [µM]
361	Hoechst 33342·3HCl (BisBenzimide)	29.66
366	Ionomycin	24.55
367	IBMX	74.99
368	NSC-95397	53.70
369	Juglone	95.70
370	K252A	3.57
374	Furoxan	89.05
379	BAY 11-7082	80.42
382	LY-294002	54.23
383	LY-83583	66.60
384	5-Iodotubercidin	42.50
385	Manoalide	40.01
386	Manumycin A	30.27
389	Gliotoxin	51.06
394	Mitomycin C	49.85
395	ML7	40.04
396	ML9	51.31
397	Monastrol	57.01
399	Monensin	24.06
401	Nocodazole	55.31
402	OBAA	38.88
403	Okadaic acid	2.07
404	Oligomycin A	21.07
406	Ouabain	28.51
408	PD 98059	62.36
409	SU-4312	63.05
411	6(5H)-Phenanthridinone	85.38
412	Phorbol 12-myristate 13-acetate	27.02
417	PP1	59.24
418	Prazocin	43.47
419	Propidium iodide	24.94
421	Puromycin·2HCl	31.42
423	SP-600125	75.68
424	Quercetin·2H2O	49.27
425	Rapamycin	18.23
426	Cyclo [Arg-Gly-Asp-D-Phe-Val]	29.00
429	Roscovitine	47.02
430	Rottlerin	32.27
432	SB 203580	44.16
433	SB 202190	50.30
434	Indirubin-3'-monoxime	60.11
437	Resveratrol	73.02
439	PP2	55.23
443	Staurosporine	35.72
446	Taxol = paclitaxel	19.52

Comp. No.	Name	Screen Conc. [µM]
450	GF-109203X	40.41
451	Tyrphostin AG-825	41.93
453	TPEN	39.26
454	Trichostatin-A	55.12
455	Trifluoperazine	34.69
456	Tunicamycin	23.19
460	Tyrphostin 9	59.02
462	U-0126	43.80
463	Valinomycin	15.00
464	Vinblastine	18.33
466	Wortmannin	38.90
469	Z-Leu3-VS	30.21
470	ZM336372	42.80
474	Mycophenolic acid	57.40
475	Shikonin	57.81
478	Cerulenin	74.65
480	Wiskostatin	39.11

331 non-toxic compounds (less than 20% cytotoxicity)

Comp. No.	Name	Screen Conc. [µM]
1	C16 Ceramide	3.33
2	C8 Ceramine	3.33
3	(R)-(+)-Methandamide	3.33
4	1α,25-Dihydroxyvitamin D3	3.33
5	1,2-Didecanoyl-glycerol (10:0)	3.33
6	1,2-Dioctanoyl-SN-glycerol	3.33
7	1,2-Dioleoyl-glycerol (18:1)	3.33
8	(±)11(12)-Epoxyeicosatrienoic acid	0.33
9	12(R)-HETE	0.33
10	12(S)-HETE	0.33
11	12(S)-HPETE	0.33
12	12-Methoxydodecanoic acid	3.33
13	13(S)-HODE	0.33
14	13(S)-HPODE	0.33
17	13-Keto-octadeca-9Z,11E-dienoic acid	0.33
18	(±)14,15-Epoxyeicosa-5Z,8Z,11Z-trienoic acid	0.33
19	15(S)-HETE	0.33
20	15(S)-HPETE	0.33
21	15-deoxy-Prostaglandin J2	3.33
22	15-Ketoicosatetraenoic acid	0.33
25	17-Phenyl-trinor-prostaglandin E2	3.33
26	1-Acyl-PAF	3.33
27	1-Hexadecyl-2-arachidonoyl-glycerol	3.33
28	1-Hexadecyl-2-methylglycero-3 PC	3.33
29	1-Hexadecyl-2-O-acetyl-glycerol	3.33
30	1-Hexadecyl-2-O-methyl-glycerol	3.33

Comp. No.	Name	Screen Conc. [µM]
32	1-Oleoyl 2-acetyl-glycerol	3.33
33	1-Stearoyl-2-lineoyl-glycerol	3.33
34	1-stearoyl-2-arachidonyl-glycerol	3.33
35	24,25-Dihydroxyvitamin D3	3.33
36	25-Dihydroxyvitamin D3	3.33
37	2-Arachidonoylglycerol	3.33
38	2-Fluoropalmitic acid	3.33
39	2-Hydroxymyristic acid	3.33
40	4-hydroxyphenylretinamide	3.33
41	4-Oxatetradecanoic acid	3.33
42	5(S)-HETE	0.33
43	5(S)-HPETE	0.33
44	5,6-Epoxyeicosatrienoic acid	0.33
45	5,8,11,14-Eicosatetraynoic acid	3.33
46	5,8,11-Eicosatriynoic acid	3.33
47	5-Ketoeicosatetraenoic acid	0.33
49	6-Keto-prostaglandin F1a	3.33
50	7,7-Dimethyleicosadienoic acid	3.33
51	8,9-Epoxyeicosatrienoic acid	0.33
52	8-epi-Prostaglandin F2a	3.33
53	9(S)-HODE	0.33
54	9(S)-HPODE	0.33
55	9,10-Octadecenoamide	3.33
56	9a,11b-Prostaglandin F2	3.33
59	Dimethyloxaloylglycine	3.33
60	Adrenic acid (22:4, n-6)	3.33
61	N-Acetyl-S-geranyl-L-cysteine	3.33
62	N-acetyl-S-geranylgeranyl-L-Cysteine	3.33
63	AM-251	3.33
65	Anandamide (18:2,n-6)	3.33
66	Anandamide (20:3,n-6)	3.33
67	Anandamide (20:4, n-6)	3.33
68	Anandamide (22:4,n-6)	3.33
69	Arachidonamide	3.33
70	Arachidonic acid (20:4, n-6)	3.33
71	Arachidonoyl-PAF	3.33
72	BML-190	3.33
73	C2 Ceramide	3.33
74	C2 Dihydroceramide	3.33
75	C8 Ceramide	3.33
76	C8 Dihydroceramide	3.33
77	Carbacyclin	3.33
78	Ciglitazone	3.33
79	Clofibrate	3.33
80	Cloprostenol	3.33

Comp. No.	Name	Screen Conc. [µM]
82	D12-Prostaglandin J2	3.33
83	Dihomo-gamma-linolenic acid	3.33
84	Dihydrosphingosine	3.33
85	Diidolylmethane	3.33
86	DL-Dihydrosphingosine	3.33
87	DL-PDMP	3.33
88	DL-PPMP	3.33
89	Docosahexaenoic acid(22:6 n-3)	3.33
90	Docosapentaenoic acid	3.33
91	Docosatrienoic acid (22:3 n-3)	3.33
92	Eicosa-5,8-dienoic acid (20:2 n-12)	3.33
93	Eicosadienoic acid (20:2 n-6)	3.33
94	Eicosapentaenoic acid (20:5 n-3)	3.33
95	Eicosatrienoic acid (20:3 n-3)	3.33
96	Enantio-PAF C16	3.33
97	Farnesylthioacetic acid	3.33
98	Fluprostenol	3.33
99	Gamma-linolenic acid (18:3 n-6)	3.33
100	Leukotoxin A (9,10-EODE)	0.33
101	Leukotoxin B (12,13-EODE)	0.33
102	Leukotriene B4	0.33
103	Leukotriene C4	0.33
104	Leukotriene D4	0.33
105	Leukotriene E4	0.33
106	Linoleamide	3.33
107	Linoleic acid	3.33
108	Linolenic acid (18:3 n-3)	3.33
109	Lipoxin A4	0.33
110	L-NASPA	3.33
111	LY-171883	3.33
112	Lyso-PAF C16	3.33
113	Lysophosphatidic acid·Na	3.33
114	D-erythro-MAPP	3.33
115	L-erythro-MAPP	3.33
116	Mead acid (20:3 n-9)	3.33
117	Mead ethanolamide	3.33
118	Methoprene acid	3.33
120	N,N-Dimethylsphingosine	3.33
121	N-Acetyl-leukotriene E4	0.33
122	N-arachidonoylglycine	3.33
123	N-linoleoylglycine	3.33
124	PAF C16	3.33
125	PAF C18	3.33
126	PAF C18:1	3.33
127	Palmitylethanolamide	3.33

Comp. No.	Name	Screen Conc. [µM]
128	Dipalmitoylphosphatidic acid	3.33
129	Prostaglandin A1	3.33
130	Prostaglandin A2	3.33
131	Prostaglandin B1	3.33
132	Prostaglandin B2	3.33
133	Prostaglandin D2	3.33
136	Prostaglandin F1a	3.33
137	Prostaglandin F2a	3.33
138	Prostaglandin I2	3.33
139	Prostaglandin J2	3.33
141	REV-5901	3.33
142	S-farnesyl-L-cysteine	3.33
143	Sphingosine	3.33
144	SQ-29548	3.33
145	TETRAHYDROCANNABINOL-7-OIC ACID	3.33
146	TTNPB	3.33
147	U-46619	3.33
148	U-75302	0.33
149	WIN 55,212-2 mesylate	3.33
150	WY-14643	3.33
151	4-Aminopyridine	177.09
152	5-Hydroxydecanoate	88.53
153	Aconitine	25.81
154	AM 92016·HCl	34.45
155	Amantadine·HCl	88.79
156	Amiloride·HCl	62.64
159	(R)-(+)-BAY K-8644	46.78
160	Benzamil·HCl	46.79
162	Cyclopiazonic acid	49.55
163	Dantrolene	53.04
164	Diazoxide	72.25
166	Diltiazem·HCl	36.96
167	E-4031	41.51
168	Fipronil	38.13
169	Flecainide acetate	35.21
170	Flufenamic acid	59.26
173	FPL-64176	47.97
174	6-Gingerol	56.62
175	Glipizide	37.41
176	Glyburide	33.74
177	Grayanotoxin III	44.99
178	R(+)-IAA-94	46.66
179	L-cis-Diltiazem·HCl	36.96
180	Lidocaine·HCl·H2O	57.71
182	Methoxyverapimil·HCl	31.98

Comp. No.	Name	Screen Conc. [µM]
183	Minoxidil	79.65
184	Minoxidil sulfate	57.61
185	Nicardipine·HCl	32.30
187	Niflumic acid	59.06
189	Nimodipine	39.83
190	Nitrendipine	46.25
191	N-Phenylanthranilic acid	78.16
192	NPPB	55.50
193	NS-1619	46.01
195	PCO-400	55.68
198	Phentolamine	59.24
199	Phenytoin	66.07
201	Pinacidil	67.94
202	Procainamide	70.82
204	Quinidine·HCl·H2O	43.99
205	Quinine·HCl·2H2O	41.99
206	QX-314	48.55
207	Ryanodine	33.77
212	TMB-8	42.14
213	Tolazamide	53.52
214	Tolbutamide	61.65
215	U-37883A	48.24
216	U-50488	45.13
217	Verapimil	36.66
218	Veratridine	24.74
219	YS035	46.37
220	ZM226600	44.64
221	Tosyl-Phe-CMK (TPCK)	47.37
222	Thiorphan	65.79
223	Z-prolyl-prolinal	50.45
226	Boc-GVV-CHO	46.63
227	Bestatin	54.05
229	MDL-28170	43.58
231	2-methoxyantimycin A3	31.18
232	HA14-1	40.73
233	Decylubiquinone	51.69
235	Deprenyl	88.99
236	1400W	94.03
237	Fumonisin B1	23.09
247	bezafibrate	46.06
248	Cycloheximide-N-ethylethanoate	45.36
249	Ascomycin (FK-520)	21.04
250	U-74389G	27.29
251	Ebselen	60.79
252	TRIM	78.55

Comp. No.	Name	Screen Conc. [µM]
254	Zaprinast	61.44
255	Vinpocetine	47.56
256	Trequinsin	41.10
257	Siguazodan	58.62
258	Rolipram	60.53
259	Ro 20-1724	59.88
260	MY-5445	50.23
261	A-3	58.53
262	Acetyl (N)-s-farnesyl-l-cysteine	45.35
264	Arvanil	37.91
266	AG213 (Tyrphostin 47)	75.67
267	AG-370	64.28
268	AG-490	56.63
272	Milrinone	78.91
273	PRIMA-1	89.98
275	4-Amino-1,8-naphthalimide	78.54
276	3-aminobenzamide (3-ABA)	122.41
281	8-methoxymethyl-IBMX	62.59
282	MBCQ	53.12
283	B581	35.41
284	BADGE	48.96
285	Bafilomycin A1	2.68
287	Blebbistatin	57.01
288	Bongkrekic acid	3.43
291	Bromo-7-nitroindazole	68.86
292	8-Bromo-cAMP	40.84
293	8-Bromo-cGMP	39.11
294	Bumetanide	45.74
295	5'-N-Ethylcarboxamidoadenosine (NECA)	54.06
296	BW-B 70C	52.69
297	CA-074-Me	41.93
298	SB-415286	46.33
299	Calpeptin	45.98
304	E-Capsaicin	54.57
305	Capsazepine	44.22
306	Castanospermine	88.09
307	Dipyridamole	33.03
310	CAPE	58.62
311	Cimaterol	76.01
312	CinnGEL	32.14
313	Cirazoline	77.06
314	Clonidine	72.43
315	Clozapine	51.00
319	Cypermethrin	40.04
320	Cytochalasin B	34.75

Comp. No.	Name	Screen Conc. [µM]
321	Cytochalasin D	32.83
322	D609	79.24
325	Decoyinine	59.68
326	1-Deoxymannojirimycin hydrochloride	83.49
327	1-Deoxynojirimycin	102.14
329	Dibutyrylcyclic AMP	33.92
330	Dibutyrylcyclic GMP	34.34
332	6,7-ADTN	93.00
335	2,5-Ditertbutylhydroquinone	74.97
338	MnTBAP	18.96
339	E6 berbamine	21.99
340	E-64-d	48.67
341	EHNA	60.09
342	Pregnenolone-16α-carbonitrile	48.81
350	GM6001	42.90
352	Nimesulide	54.06
355	H9	51.40
356	HA1077	45.75
357	HA-1004	56.82
358	HBDDE	49.26
359	Histamine	149.95
360	HNMPA-(AM)3	36.68
362	(-)-Huperzine A	68.78
363	24(S)-hydroxycholesterol	41.39
364	ICRF-193	59.04
365	Indomethacin	46.58
371	KN-62	23.09
372	KT-5720	3.94
373	L-744,832	29.77
375	Latrunculin B	42.14
376	Lavendustin A	43.70
377	N9-Isopropylolomoucine	51.06
378	Leupeptin	39.07
380	Tyrphostin-8	97.94
381	L-NAME	71.46
387	Mastoparan	9.87
388	MCI-186	95.68
390	LFM-A13	46.30
391	Methotrexate	39.27
392	(±)-Epibatidine	79.86
393	Mevinolin (Lovastatin)	41.20
398	GW-5074	31.99
400	FK-506	20.73
405	Olomoucine	55.86
407	PCA 4248	46.11

Comp. No.	Name	Screen Conc. [µM]
410	Pepstatin	24.30
413	Pifithrin-α	58.20
414	CITCO	38.16
415	Piroxicam	50.30
416	Cyclopamine	40.49
420	(S)-(-)-propranolol·HCl	56.34
422	SB-431542	43.36
427	RHC-80267	42.25
428	RK-682	45.23
431	RWJ-60475-(AM)3	26.87
435	Indirubin	63.55
436	Serotonin	94.58
438	Splitomycin	84.08
440	Piceatannol	68.24
441	SQ22536	81.22
442	Alrestatin	65.30
444	Swainsonine	96.22
445	Tamoxifen	44.86
447	RG-14620	60.58
448	Thalidomide	64.54
449	Thiocitrulline [L-Thiocitrulline]	87.15
452	Kavain (+/-)	72.38
457	Nafamostat mesylate	37.58
458	Tyrphostin AG-126	77.46
459	Tyrphostin 1	90.48
461	U73122	35.87
465	W7	44.17
467	Y-27632	67.38
468	yohimbine	42.64
471	zVAD-FMK	0.02
472	NS-398	53.02
473	AA-861	51.06
476	2-APB	74.04
477	CDC	51.87
479	Phenoxybenzamine	54.86

Table S1. Assessment of compound toxicity to THP-1 cells. Table represents 149 toxic (20%<cytotoxicity) and 331 non-toxic (20%>cytotoxicity) compounds at indicated concentration as assessed by the MTT assay.

Supplementary Table S2. Twenty-one compounds that inhibit infection by *B. abortus*.

Compound	Description	CIDs	PRE	POST
**Kinase signaling**				
**A-3**	Kinase Inhibitor	9861903	99	80
**AG-213 (Tyrphostin 47)**	EGF-R tyrosine kinase inhibitor	5485187	70	64
**H9***	Kinase Inhibitor	11957465	96	71
**LFM-A13**	BTK inhibitor	54676905	100	80
**Ion homeostasis**				
**2-APB***	IP3 receptor blocker	1598	98	80
**NPPB***	Miscelaneous channels	4549	81	80
**Nicardipine·HCl**	Calcium channels	41114	69	62
**Arvanil**	Vaniloid receptor agonist	6449767	71	73
**W-7**	Calmodulin antagonist	124887	91	75
**Kavain (+/-)**	Voltage-dependent Na channel inhibitor	5369129	107	71
**Nitric oxide**				
**1400W**	iNOS inhibitor	2733515	105	78
**L-NAME**	NOS inhibitor	135193	62	63
**Protease Inhibitors**				
**MDL-28170**	Calpain inhibitor	72430	103	63
**Z-prolyl-prolinal**	Prolyl endopeptidase inhibitor	122623	87	70
**Other**				
**HA14-1**	Bcl-2 ligand induces apoptosis	3549	103	75
**CAPE**	Antioxidant/ NFkappa B inhibitor	5281787	102	74
**AA-861**	5-lipoxygenase inhibitor	1967	81	44
**Splitomycin**	Sir2p inhibitor	5269	73	70
**3-aminobenzamide (3-ABA)**	ADP ribose polymerase, apoptosis inhibitor	1645	91	71
**Acetyl (N)-s-farnesyl-l-cysteine**	Farnesylation inhibitor	1994	87	65
**EHNA**	PDE2 inhibitor/adenosine deaminase inhibitor	11957547	76	97

Table S2. Twenty-one compounds that inhibit infection by *B. abortus.* Non-cytotoxic compounds representing 18 host-specific and three compounds that overlapped with *Brucella* metabolic inhibitors (denoted with asterisks) fall into five functional categories. Each compound has a corresponding PubChem Compound Identified (CID) and intracellular inhibition efficiencies, calculated as a percentage of intracellular mCherry signal normalized to untreated pre- (PRE) and post-infection (POST) controls.

Supplementary Table S3. Microscopy-based cytotoxocity of compounds that inhibit infection by *B. abortus*

non-toxic (green) | moderately-toxic (yellow) | toxic (red)

Compound	Description	CIDs	Cytotoxicity Nuclei	Cytotoxicity StDev	Metabolic Inh.	Canonical SMILES
**Posttranslational modifications**						
**EHNA**	PDE2 inhibitor/adenosine deaminase inhibitor	11957547	65.0	12.9	NO	C1=CC2=C(C=CN=C2)C(=C1)S(=O)(=O)NCCN.Cl.Cl
**A-3**	Kinase Inhibitor	9861903	57.6	11.4	NO	C1=CC2=C(C=CC=C2Cl)C(=C1)S(=O)(=O)NCCN.Cl
**AG213 (Tyrphostin 47)**	EGF-R tyrosine kinase inhibitor	5485187	47.7	10.7	NO	C1=CC(=O)C(=CC1=CC(=C(N)S)C#N)O
**H9**	Kinase Inhibitor	11957465	46.5	10.7	YES	C1=CC2=C(C=CN=C2)C(=C1)S(=O)(=O)NCCN.Cl.Cl
**LFM-A13**	BTK inhibitor	9549280	42.9	10.5	NO	CC(=C(C#N)C(=O)NC1=C(C=CC(=C1)Br)Br)O
**AG-879**	Tyrosine kinase inhibitor	5487525	37.7	10.7	YES	CC(C)(C)C1=CC(=CC(=C(N)S)C#N)C=C(C1=O)C(C)(C)C
**Tyrphostin 9**	PDGF-R tyrosine kinase inhibitor	5614	25.5	5.7	YES	CC(C)(C)C1=CC(=CC(=C1O)C(C)(C)C)C=C(C#N)C#N
**BAY 11-7082**	IKK kinase inhibitor	5353431	9.0	3.7	YES	CC1=CC=C(C=C1)S(=O)(=O)C=CC#N
**Indirubin-3'-monoxime**	GSK-3beta kinase inhibitor	5326739	1.5	2.1	YES	C1=CC=C2C(=C1)C(=C(N2)C3=C4C=CC=CC4=NC3=O)NO
**Ion homeostasis**						
**Nicardipine·HCl**	Calcium channels	41114	65.0	6.8	NO	CC1=C(C(C(=C(N1)C)C(=O)OCCN(C)CC2=CC=CC=C2)C3=CC(=CC=C3)[N+](=O)[O-])C(=O)OC.Cl
**NPPB**	Miscelaneous channels	4549	39.3	4.5	YES	C1=CC=C(C=C1)CCCNC2=C(C=C(C=C2)[N+](=O)[O-])C(=O)O
**Kavain (+/-)**	Voltage-dependent Na channel inhibitor	5369129	63.1	7.5	NO	COC1=CC(=O)OC(C1)C=CC2=CC=CC=C2
**Niflumic acid**	Misc. channels	4488	59.9	13.6	YES	C1=CC(=CC(=C1)NC2=C(C=CC=N2)C(=O)O)C(F)(F)F
**Flufenamic acid**	Potassium channels	3371	48.0	10.7	YES	C1=CC=C(C(=C1)C(=O)O)NC2=CC=CC(=C2)C(F)(F)F
**Arvanil**	Vaniloid receptor agonist	6449767	45.4	8.8	NO	CCCCCC=CCC=CCC=CCC=CCCCC(=O)NCC1=CC(=C(C=C1)O)OC
**N-Phenylanthranilic acid**	Misc. channels	4386	40.7	2.2	YES	C1=CC=C(C=C1)NC2=CC=CC=C2C(=O)O
**W-7**	Calmodulin antagonist/MLC kinase inhibitor	124887	34.6	6.3	NO	C1=CC2=C(C=CC=C2Cl)C(=C1)S(=O)(=O)NCCCCCCN.Cl
**2-APB**	IP3 receptor blocker	1598	23.7	5.4	YES	B(C1=CC=CC=C1)(C2=CC=CC=C2)OCCN
**Protease inhibitors**						
**Tosyl-Phe-CMK (TPCK)**	Serine protease inhibitor	439647	56.1	7.2	YES	CC1=CC=C(C=C1)S(=O)(=O)NC(CC2=CC=CC=C2)C(=O)CCl
**Z-prolyl-prolinal**	Prolyl endopeptidase inhibitor	122623	59.9	22.1	NO	C1CC(N(C1)C(=O)C2CCCN2C(=O)OCC3=CC=CC=C3)C=O
**MDL-28170**	Calpain inhibitor	72430	38.9	4.3	NO	CC(C)C(C(=O)NC(CC1=CC=CC=C1)C=O)NC(=O)OCC2=CC=CC=C2
**Energy metabolism**						
**Methotrexate**	DHFR inhibitor	126941	38.0	11.6	YES	CN(CC1=CN=C2C(=N1)C(=NC(=N2)N)N)C3=CC=C(C=C3)C(=O)NC(CCC(=O)O)C(=O)O
**FCCP**	Mitochondrial uncoupler	3330	17.4	7.8	YES	C1=CC(=CC=C1NN=C(C#N)C#N)OC(F)(F)F
**Diphenyleneiodonium**	Flavoprotein inhibitor	3101	0.8	0.4	YES	C1=CC=C2C(=C1)C3=CC=CC=C3[I+]2
**Nitric oxide**						
**1400W**	iNOS inhibitor	2733515	59.9	15.2	NO	CC(=NCC1=CC(=CC=C1)CN)N.Cl.Cl
**L-NAME**	NOS inhibitor	135193	48.3	7.1	NO	COC(=O)C(CCCN=C(N)N[N+](=O)[O-])N.Cl
**Farnesylation**						
**Acetyl (N)-s-farnesyl-l-cysteine**	Farnesylation inhibitor	1994	27.0	6.2	NO	CC(=CCCC(=CCCC(=CCSCC(C(=O)O)NC(=O)C)C)C)C
**Manumycin A**	Ras farnesylation inhibitor	6438330	14.7	3.8	YES	CCCCC(C)C=C(C)C=C(C)C(=O)NC1=CC(C2C(C1=O)O2)(C=CC=CC=CC(=O)NC3=C(CCC3=O)O)O
**Metal chelators**						
**Hinokitiol**	Iron chelator	3611	11.2	2.9	YES	CC(C)C1=CC(=O)C(=CC=C1)O
**TPEN**	Heavy metal chelator	5519	2.7	2.4	YES	C1=CC=NC(=C1)CN(CCN(CC2=CC=CC=N2)CC3=CC=CC=N3)CC4=CC=CC=N4
**DNA modification**						
**Mitomycin C**	Cross links DNA	5746	14.5	4.0	YES	CC1=C(C(=O)C2=C(C1=O)N3CC4C(C3(C2COC(=O)N)OC)N4)N
**beta-lapachone**	Topoisomerase 1 inhibitor	3885	1.6	1.8	YES	CC1(CCC2=C(O1)C3=CC=CC=C3C(=O)C2=O)C
**Lipid biosynthesis**						
**Cerulenin**	Fatty acid biosynthesis inhibitor	5282054	6.3	4.1	YES	CC=CCC=CCCC(=O)C1C(O1)C(=O)N
**CDC**	12-Lipoxygenase inhibitor	9905190	5.8	2.0	YES	C1=CC=C(C=C1)C=CCOC(=O)C(=CC2=CC(=C(C=C2)O)O)C#N
**Other**						
**Splitomycin**	Sir2p inhibitor	5269	52.3	4.4	NO	C1CC(=O)OC2=C1C3=CC=CC=C3C=C2
**HA14-1**	Bcl-2 ligand induces apoptosis	3549	52.1	11.3	NO	CCOC(=O)C1=C(OC2=C(C1C(C#N)C(=O)OCC)C=C(C=C2)Br)N
**3-aminobenzamide (3-ABA)**	ADP ribose polymerase, apoptosis inhibitor	1645	46.6	8.2	NO	C1=CC(=CC(=C1)N)C(=O)N
**CAPE**	Antioxidant/ NFkappa B inhibitor	5281787	33.3	5.9	NO	C1=CC=C(C=C1)CCOC(=O)C=CC2=CC(=C(C=C2)O)O
**AA-861**	5-lipoxygenase inhibitor	1967	22.9	4.8	NO	CC1=C(C(=O)C(=C(C1=O)C)CCCCC#CCCCC#CCO)C
**NSC-95397**	CDC25 phosphatase inhibitor	262093	8.4	4.8	YES	C1=CC=C2C(=C1)C(=O)C(=C(C2=O)SCCO)SCCO
**Nigericin**	Induces intracellular acidification	34230	3.3	1.5	YES	CC1CCC(OC1C(C)C(=O)O)CC2CC(C(C3(O2)C(CC(O3)(C)C4CCC(O4)(C)C5C(CC(O5)C6C(CC(C(O6)(CO)O)C)C)C)C)C)OC
**Juglone**	PIN1 inhibitor	3806	2.8	1.9	YES	C1=CC2=C(C(=O)C=CC2=O)C(=C1)O
**LY-83583**	Inhibits NO-activation of guanylate cyclase	3976	2.2	0.8	YES	C1=CC=C(C=C1)NC2=CC(=O)C3=C(C2=O)C=CC=N3
**Wiskostatin**	N-WASP inhibitor	2775510	1.7	1.2	YES	CN(C)CC(CN1C2=C(C=C(C=C2)Br)C3=C1C=CC(=C3)Br)O

Table S3. Microscopy-based assessment of cytotoxicity of compounds that inhibit infection by *B. abortus*. Forty-four compounds: 26 *Brucella*-targeting compounds are indicated as "YES" in the metabolic inhibitors (Metabolic inh.) column and 18 host-targeting compounds are indicated as "NO". Each compound has a corresponding PubChem Compound Identified (CID), cytotoxicity represented as an average nuclei count (Nuclei) and corresponding standard deviation (StDev) calculated from 6-24 images per treatment, and the corresponding canonical **S**implified **M**olecular-**i**nput **l**ine-**e**ntry **s**ystem (SMILES) string obtained from PubChem Database. Compounds that had a significantly lower average number of nuclei than control and led to cytotoxicity-induced morphological changes in THP-1 cells were deemed toxic and are highlighted red. Compounds that showed moderate cytotoxicity but did not lead to morphological changes in THP-1 cells are highlighted yellow. Non-toxic compounds are highlighted green and represent compounds that did not affect nuclei count and did not induce morphological changes in THP-1 cells relative to control.

Figure S1. Qualitative assessment of cell morphology.

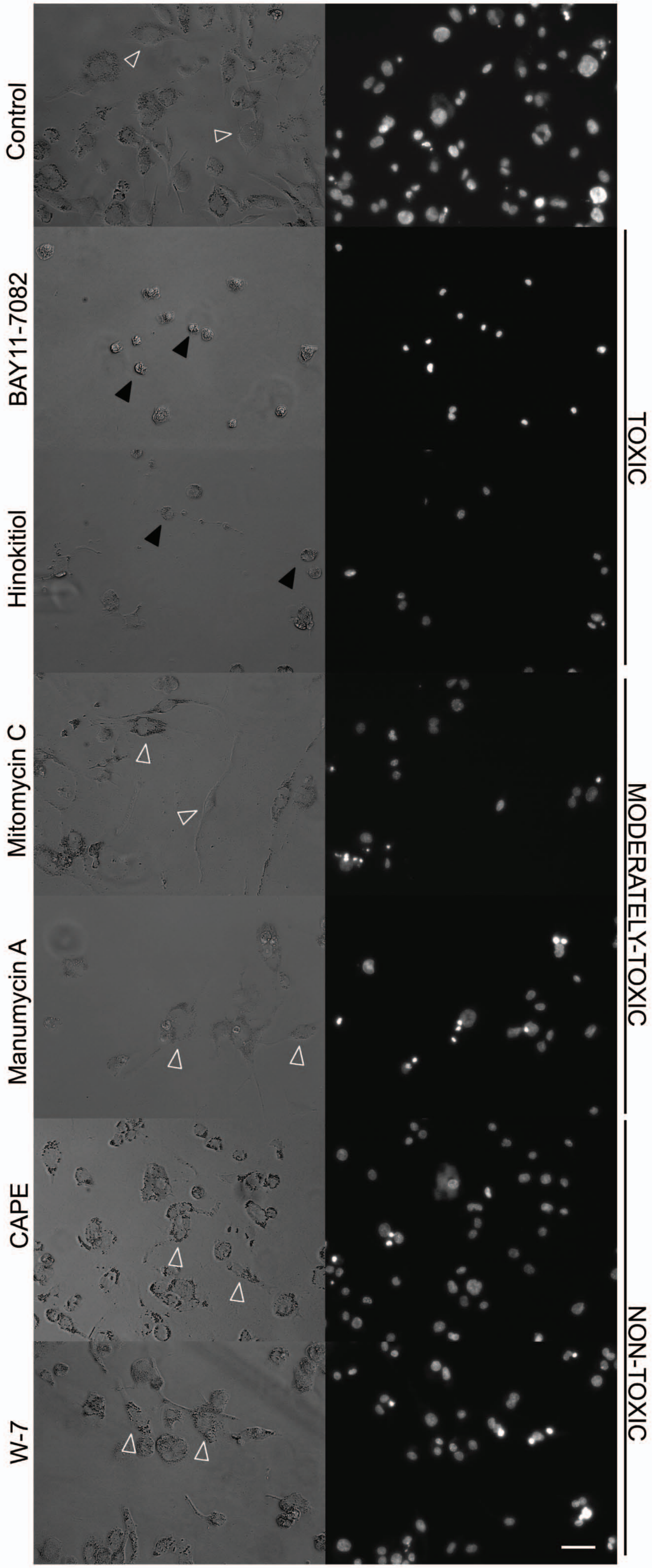

Figure S1. Qualitative assessment of cell morphology. Forty-four compounds that inhibited *Brucella* infection (18 host-targeting and 26 *Brucella*-targeting) were imaged to assess changes in cell morphology. Shown two representative compounds, BAY11-7082 and hinokitiol, were toxic based on a distinct morphological damage to the cells. These cells were marked as toxic. Two representatives, mitomycin C and manumycin A, were shown to decrease nuclei count, but the cells were morphologically similar to controls. Such cells were marked as "moderately-toxic" and were retained as final hits. Two representative compounds, CAPE and W-7, were non-cytotoxic as assessed by both nuclei count and imaging, which showed cellular morphology to be similar to control cells. These cells were marked as "non-toxic". The opened triangles indicate examples of cells with wild-type morphology. Filled triangles indicate examples of cells with significant morphological changes exhibiting rounding and shrinkage. Scale bar = 50 µm.

Figure S2. Analysis of chemical structures similarities of *Brucella*-targeting compounds.

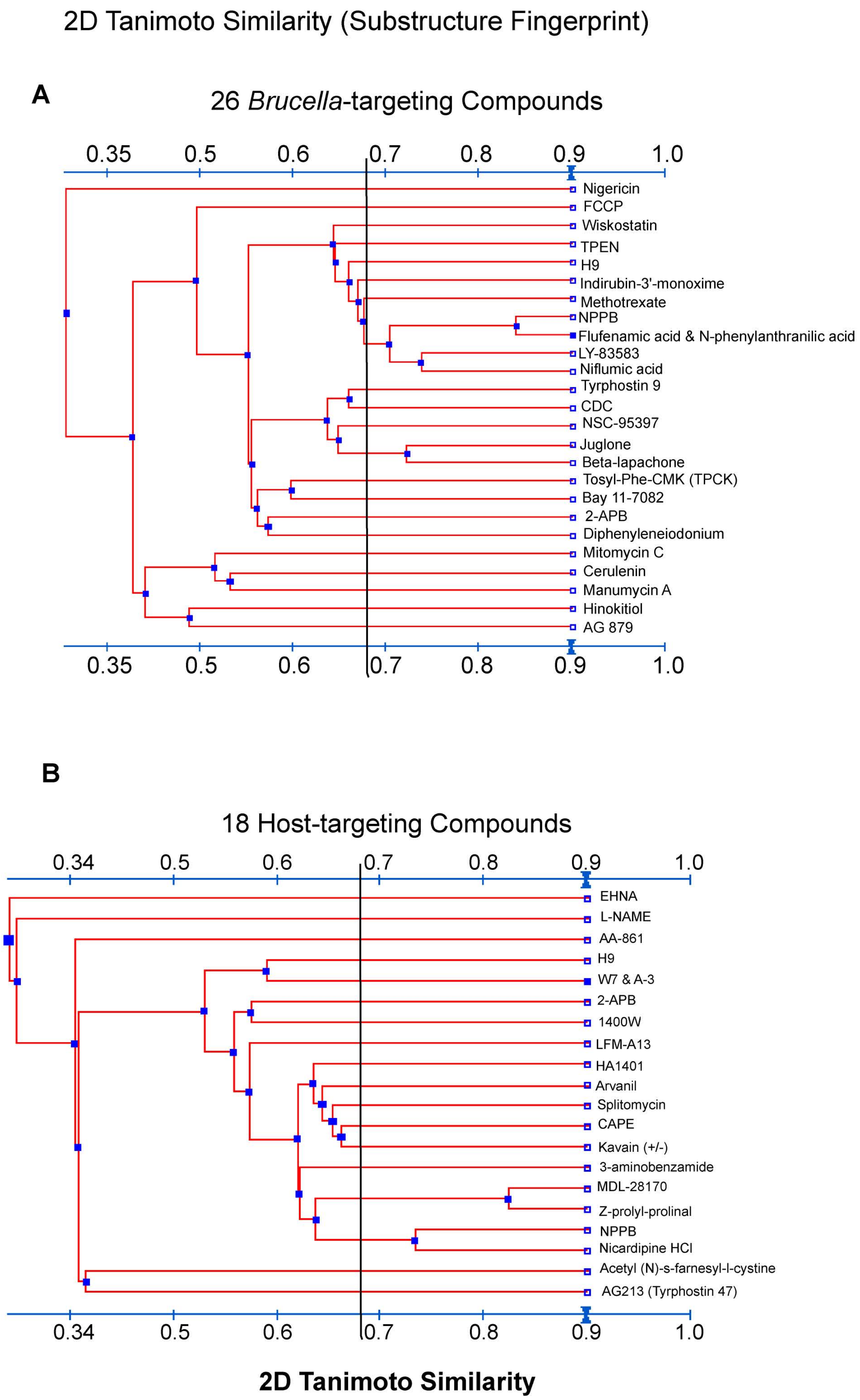

Figure S2. Analysis of chemical structures similarities of *Brucella*-targeting compounds. Chemical structure similarities between **A)** 26 compounds that inhibit *B. abortus* metabolism in axenic culture and **B)** 18 host-targeting hit compounds identified in the initial fluorescence-based screen. Three of the compounds (H9, NPPB, and 2-APB) overlap between these two datasets. The Tanimoto Similarity Score was generated using PubChem Structure Clustering Tool. A Tanimoto Similarity Score of >0.68 (black vertical line) is statistically significant.

Figure S3. Compound structures.

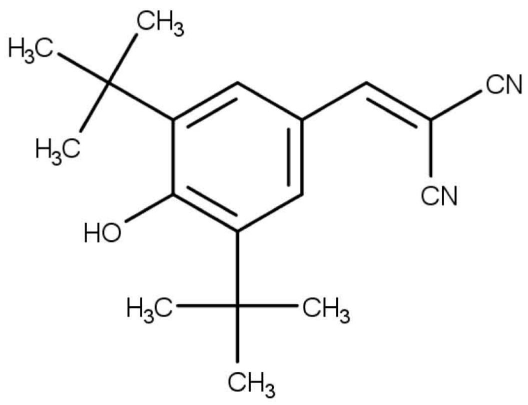

Tyrphostin 9, CID: 5614

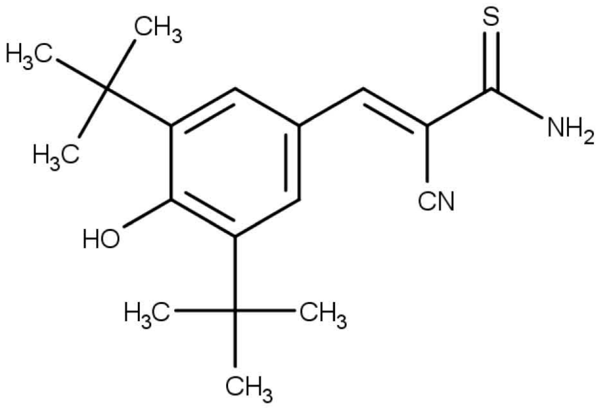

AG-879, CID: 5487525

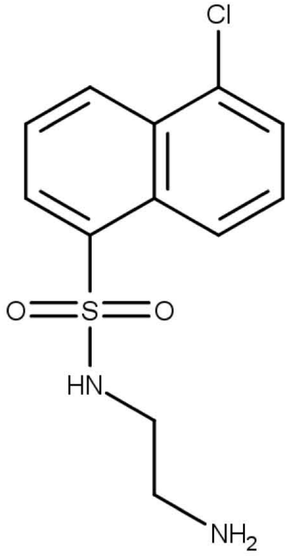

A-3, CID: 9861903

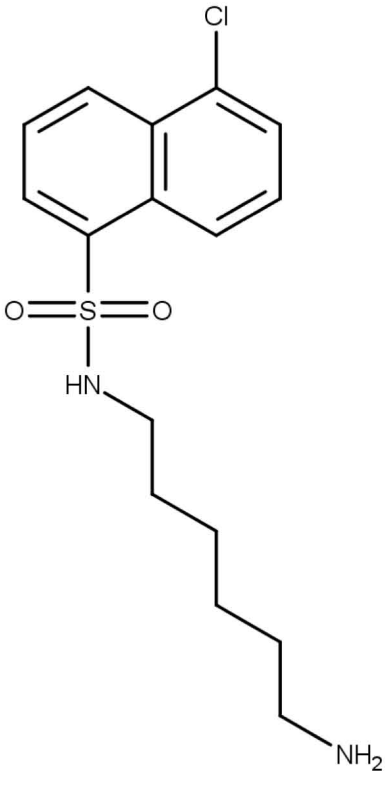

W7, CID: 124887

Figure S3. Compound structures. Examples of two pairs of structurally related compounds with their corresponding PubChem Compound Identifiers (CIDs). Structures were generated using MarvinSketch 16.1.25.0, 2016, ChemAxon (http://www.chemaxon.com)

Figure S4. Quantification of intracellular *B. abortus* at dose-response.

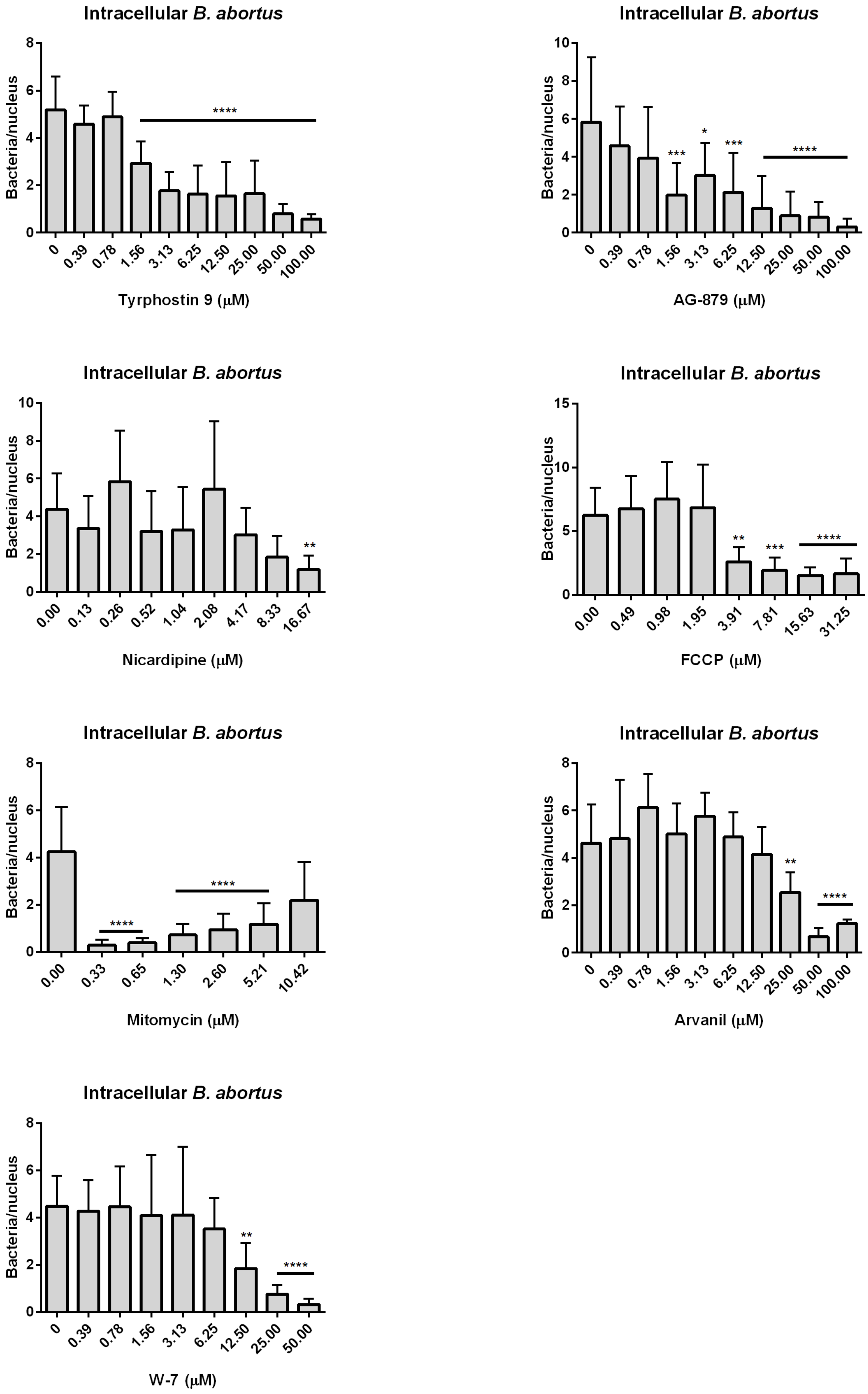

Figure S4. Quantification of intracellular *B. abortus* at dose-response.

Quantification of compound efficiency by measuring the average ratio of bacteria-to-nucleus in THP-1 cells treated with selected final candidate compounds. Graph represent selected dataset out of four independent replicates. Ten images were used for quantification of bacteria-to-nucleus ratio per each replicate. Statistical significance was evaluated using one-way ANOVA followed by Dunnett's test (*$p<0.05$, **$p<0.01$, ***$p<0.001$, ****$p<0.0001$). Error bars represent +/- standard deviation.
